# A new perspective on the regulation of neuroinflammation in intracerebral hemorrhage: mechanisms of NLRP3 inflammasome activation and therapeutic strategies

**DOI:** 10.3389/fimmu.2025.1526786

**Published:** 2025-02-27

**Authors:** Kai-long He, Xian Yu, Lei Xia, Yan-dong Xie, En-bo Qi, Liang Wan, Xu-ming Hua, Chao-hui Jing

**Affiliations:** ^1^ Department of Neurosurgery, XinHua Hospital, Affiliated to Shanghai JiaoTong University School of Medicine, Shanghai, China; ^2^ Department of Neurosurgery, The Second Affiliated Hospital of Zhejiang University School of Medicine, Hangzhou, China

**Keywords:** intracerebral hemorrhage, neuroinflammation, NLRP3 inflammasome, microglia, astrocyte, inflammation regulation, clinical translation

## Abstract

Intracerebral hemorrhage (ICH), a specific subtype within the spectrum of stroke disorders, is characterized by its high mortality and significant risk of long-term disability. The initiation and progression of neuroinflammation play a central and critical role in the pathophysiology of ICH. The NOD-like receptor family pyrin domain-containing 3 (NLRP3) inflammasome, a protein complex involved in initiating inflammation, is the central focus of this article. Microglia and astrocytes play critical roles in the inflammatory damage process associated with neuroinflammation. The NLRP3 inflammasome is expressed within both types of glial cells, and its activation drives these cells toward a pro-inflammatory phenotype, which exacerbates inflammatory damage in the brain. However, the regulatory relationship between these two cell types remains to be explored. Targeting NLRP3 inflammasomes in microglia or astrocytes may provide an effective approach to mitigate neuroinflammation following ICH. This article first provides an overview of the composition and activation mechanisms of the NLRP3 inflammasome. Subsequently, it summarizes recent research findings on novel signaling pathways that regulate NLRP3 inflammasome activity. Finally, we reviewed recent progress in NLRP3 inflammasome inhibitors, highlighting the clinical translation potential of certain candidates. These inhibitors hold promise as innovative strategies for managing inflammation following ICH.

## 1 Introduction

Intracerebral hemorrhage(ICH) results from the rupture of blood vessels and is a severe subtype of stroke, characterized by the infiltration of blood into the brain tissue (1, 2). In all stroke cases, ICH accounts for approximately 10% to 15%, and it is associated with a high incidence and mortality rate (3). Primary brain injury triggered by ICH mainly refers to the space-occupying effect of the hematoma that forms after the rupture of cerebral blood vessels. This effect leads to a marked increase in intracranial pressure, which subsequently results in a gradual deterioration of neurological function (4). Secondary brain injury following ICH is mediated by blood and its metabolic byproducts, with the pathological processes encompassing inflammatory responses, mitochondrial dysfunction, oxidative stress, neuronal apoptosis, and disruption of the blood-brain barrier (BBB) (5, 6). Hematoma evacuation surgery is the primary treatment for ICH in neurosurgery. While this procedure can reduce morbidity associated with the surgery, it does not mitigate secondary brain injury resulting from the ICH (3, 7). Neuroinflammation is a primary driver of secondary brain injury following ICH (8). Thus, inhibiting neuroinflammation is a crucial strategy for improving the prognosis of ICH (9).

Neuroinflammation is defined as the robust host defense response immediately initiated when blood enters the brain parenchyma (6). After ICH, a cascade of immune cell activations initiates neuroinflammation. Among these immune responses, microglia play a particularly pivotal role, acting as central drivers that exacerbate inflammatory injury (9). The activation of microglia and other immune cells results in the release of pro-inflammatory cytokines, including tumor necrosis factor (TNF)-α, interleukin (IL)-1β, myeloperoxidase (MPO), and other cytotoxic substances (10). The sustained activation of the inflammatory cascade is maintained by pro-inflammatory cytokines, a process that leads to widespread neuronal loss, disruption of the BBB, the formation of cerebral edema, and ultimately, neurological dysfunction (6, 11, 12). Neuroinflammation increases BBB permeability, leading to edema that exacerbates the space-occupying effect, which in turn induces secondary ischemia. This ischemia accelerates cellular death and inflicts additional inflammatory damage on the surrounding brain tissue (13).

Among the different inflammasomes identified, the NOD-like receptor family pyrin domain-containing 3 (NLRP3) inflammasome has been the subject of the most extensive research (14, 15). Growing evidence indicates that NLRP3 inflammasome activation after ICH amplifies the inflammatory cascade. Suppressing NLRP3 inflammasome activity has been shown to inhibit the maturation of pro-inflammatory cytokines, thereby promoting neurological recovery and improving outcomes for ICH patients (16, 17). Consequently, targeting NLRP3 inflammasome-mediated neuroinflammation presents a compelling therapeutic strategy for minimizing inflammatory damage and enhancing recovery outcomes after ICH. This review examines the pathways by which the NLRP3 inflammasome contributes to neuroinflammation following ICH and evaluates potential therapeutic approaches to counteract its impact.

## 2 Introduction to the NLRP3 inflammasome

### 2.1 The concept of the NLRP3 inflammasome

**Figure 1:**
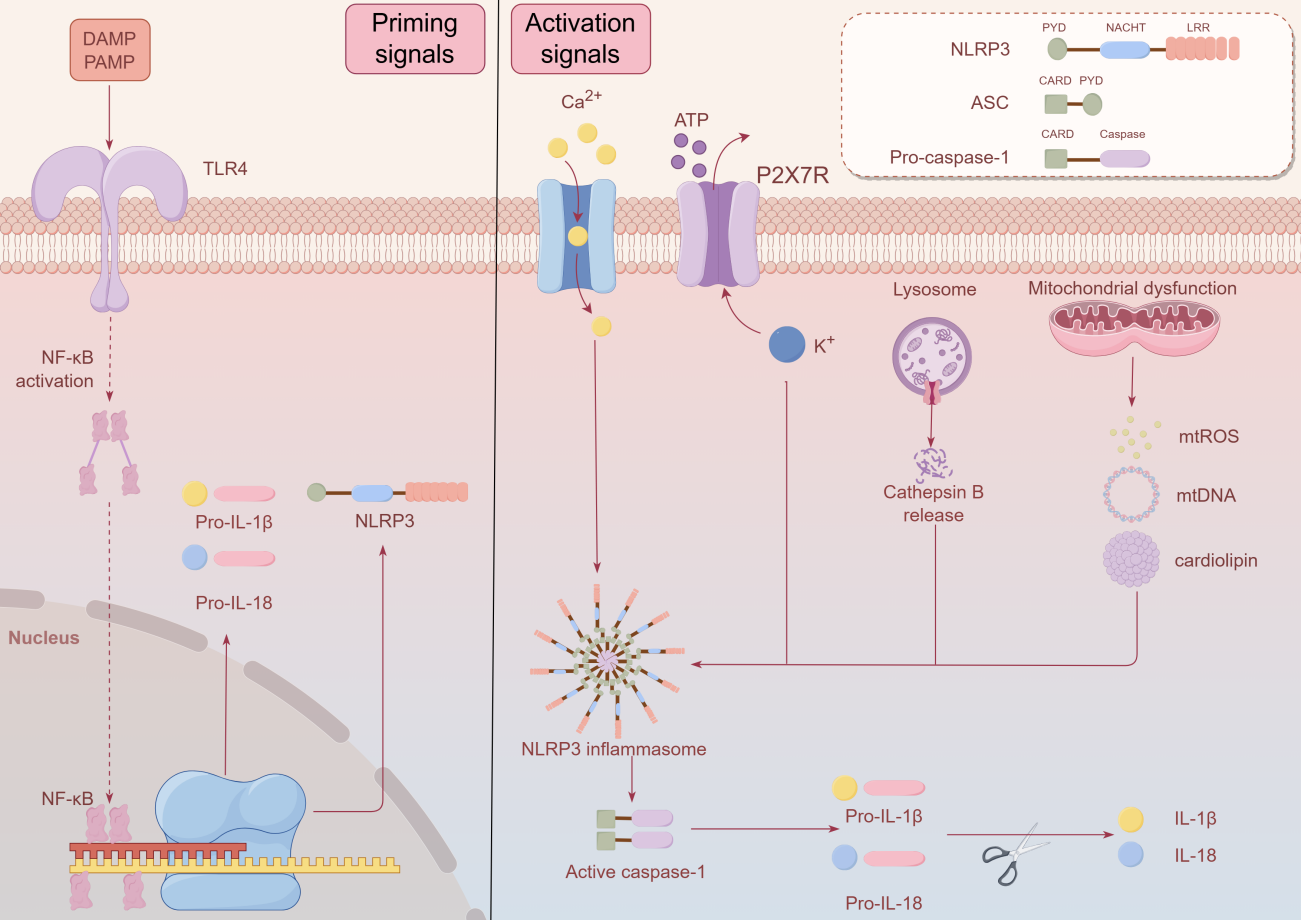
Schematic diagram of NLRP3 inflammasome composition and activation after ICH. The NLRP3 inflammasome is a multiprotein complex typically composed of the NLRP3 sensor, the ASC (apoptosis-associated speck-like protein), and the pro-caspase-1 precursor. NLRP3 is a complex multi-domain protein, consisting of three main components: the N-terminal pyrin domain (PYD), the central nucleotide-binding and oligomerization domain (NACHT), and the C-terminal leucine-rich repeat (LRR) region. ASC is a protein containing an N-terminal PYD and a C-terminal caspase recruitment domain (CARD).Pro-caspase-1 consists of two main domains: the CARD and the caspase activation domain. Upon detection of danger signals, the PYD domain of NLRP3 binds to ASC, which in turn recruits caspase-1 through its CARD domain, leading to the assembly of the NLRP3 inflammasome. In the priming signals, Toll-like receptors (TLRs) are capable of recognizing pathogen-associated molecular patterns (PAMPs) and damage-associated molecular patterns (DAMPs), which in turn trigger the nuclear factor kappa B (NF-κB) signaling pathway activation. The activation of NF-κB promotes its translocation to the nucleus, where it binds to DNA, thereby enhancing the transcriptional expression of NLRP3, pro-IL-1β, and pro-IL-18. During the Activation Signals, the NLRP3 inflammasome activation can be initiated by various stimuli, including purinergic P2X7 receptor (P2X7R)-mediated potassium efflux, intracellular calcium influx, reactive oxygen species (ROS) production caused by mitochondrial stress, the secretion of mitochondrial DNA or cardiolipin from mitochondria, and the release of cathepsin B following lysosomal rupture. Activation of the NLRP3 inflammasome promotes the autocatalytic maturation of pro-caspase-1 into active caspase-1, which then cleaves the precursors of IL-1β and IL-18 into their biologically active mature forms. This figure was created by Figdraw.

The NLRP3 inflammasome is pivotal in the development of numerous diseases, spanning neurodegenerative conditions such as multiple sclerosis, Alzheimer’s, and Parkinson’s diseases, as well as metabolic disorders like atherosclerosis, type 2 diabetes, and obesity (18). The NLRP3 inflammasome is a multiprotein complex typically composed of the NLRP3 sensor, the ASC (apoptosis-associated speck-like protein), and the pro-caspase-1 precursor (**Figure 1**) (19). NLRP3 is a complex multi-domain protein, consisting of three main components: the N-terminal pyrin domain (PYD), the central nucleotide-binding and oligomerization domain (NACHT), and the C-terminal leucine-rich repeat (LRR) region. It is one of the most widely studied members of the inflammasome family (20, 21). ASC, also known as PYCARD, is a protein containing an N-terminal PYD and a C-terminal caspase recruitment domain (CARD). It promotes the activation of caspase-1 (22). Pro-caspase-1 consists of two main domains: the CARD and the caspase activation domain (23).In the absence of immune activation signals, an autoinhibitory interaction between the NACHT domain and the LRR domain effectively prevents the binding of NLRP3 to ASC, thereby inhibiting the formation of the inflammasome (24). Upon detection of danger signals, the PYD domain of NLRP3 binds to ASC, which in turn recruits caspase-1 through its CARD domain, leading to the assembly of the NLRP3 inflammasome. This multiprotein complex activates pro-caspase-1 via autocatalytic cleavage, leading to the generation of active caspase-1. Active caspase-1 cleaves the precursor forms of the cytokines IL-1β and IL-18, producing their active forms. These activated cytokines play a crucial mediatory role in the inflammatory response (19, 24, 25).

### 2.2 Activation of the NLRP3 inflammasome

The activation of the NLRP3 inflammasome can be triggered by various stimuli, including viruses, bacteria, fungi, pore-forming toxins, crystal substances (such as urate crystals, silica, asbestos, and alum), and extracellular ATP. These factors activate the NLRP3 inflammasome through different mechanisms, initiating a cascade of intracellular reactions (26, 27). The NLRP3 inflammasome activation can be initiated by various stimuli, including purinergic P2X7 receptor (P2X7R)-mediated potassium efflux, intracellular calcium influx, reactive oxygen species (ROS) production caused by mitochondrial stress, the secretion of mitochondrial DNA or cardiolipin from mitochondria, and the release of cathepsin B following lysosomal rupture (25, 27). The activation of the inflammasome is a process involving two stages: the initial priming signals and the subsequent activation signals (**Figure 1**). In the priming signals, Toll-like receptors (TLRs) are capable of recognizing pathogen-associated molecular patterns (PAMPs) and damage-associated molecular patterns (DAMPs), which in turn trigger the nuclear factor kappa B (NF-κB) signaling pathway activation. The activation of NF-κB promotes its translocation to the nucleus, where it binds to DNA, thereby enhancing the transcriptional expression of NLRP3, pro-IL-1β, and pro-IL-18 (24, 28). Various factors, including TNF, IL-1, high-mobility group box 1 (HMGB1), interferon (IFN), transforming growth factor-β (TGF-β), and lipopolysaccharide (LPS), can promote the priming signals of NLRP3 inflammasome activation. These molecules play crucial roles in triggering the NF-κB signaling pathway, which leads to the upregulation of key inflammasome components and preparing the system for subsequent activation (29, 30). During the activation signals, NLRP3 undergoes oligomerization, forming a complex consisting of NLRP3, ASC, and pro-caspase-1. The formation of this complex promotes the autocatalytic maturation of pro-caspase-1 into active caspase-1, which then cleaves the precursors of IL-1β and IL-18 into their biologically active mature forms (31–33). Recent studies have established an important link between the NLRP3 inflammasome and the inflammatory response triggered in ICH (34). After ICH, various upstream signals can activate the NLRP3 inflammasome, with heme, released during hemoglobin degradation, playing a crucial role (35). Following ICH, the NLRP3 inflammasome activation exacerbates both the degree of cerebral edema and the neuroinflammatory response (36).

## 3 Neuroinflammation mediated by innate immune cells

The complex immune response is a critical factor in the development of neuroinflammation after intracerebral hemorrhage, involving various immune cells, among which microglia and astrocytes play pivotal roles. The NLRP3 inflammasome is expressed in both microglia and astrocytes, and numerous studies have demonstrated that the activation of the NLRP3 inflammasome in these cells can alleviate neuroinflammation under specific pathological conditions. In the following section, we focus on neuroinflammation mediated by microglia and astrocytes, as well as the potential relationship between the NLRP3 inflammasome expressed in these cells and neuroinflammation after intracerebral hemorrhage.

### 3.1 Microglia mediated neuroinflammation

Microglia play a critical immune regulatory role in the central nervous system (CNS), which is vital for both the physiological and pathological processes of neuroinflammation (37). The pathological characteristics of neuroinflammation are usually marked by an increase in pro-inflammatory cytokine expression, enhanced activation of microglia, infiltration of peripheral immune cells like leukocytes into neural tissue, and subsequent structural and functional damage to the nervous system (38). Microglia-mediated neuroinflammation is crucial in the inflammatory injury process. Upon activation, microglia transition from their branched morphology to an amoeboid shape, enhancing their phagocytic activity and reactivity (9, 39). M1-like microglia are characterized by a pro-inflammatory and neurotoxic phenotype, marked by the secretion of TNF-α, IL-1β, IL-12, and inducible nitric oxide synthase (INOS). These factors contribute to the exacerbation of neuroinflammation and the progression of tissue damage (40–42). M2 microglia secrete anti-inflammatory factors, suppress immune responses, and promote tissue repair and neuroregeneration. These functions are crucial for alleviating inflammation and aiding recovery after brain injury. They stimulate the release of anti-inflammatory cytokines, such as IL-4, IL-10, IL-13, and transforming growth factor-β (TGF-β), and are characterized by markers like arginase-1 (Arg-1) and CD206, which contribute to the repair process (40, 42). Simultaneously, M2 microglia facilitate axonal regeneration and vascular remodeling by secreting remodeling factors such as vascular endothelial growth factor (VEGF), brain-derived neurotrophic factor (BDNF), which play crucial roles in tissue repair and neurovascular recovery (43). Furthermore, during the remodeling phase following a stroke, microglia and infiltrating macrophages function as essential phagocytic cell populations, collaborating to clear dead cells and tissue debris, thus facilitating the repair and reconstruction of damaged neural tissues (44). ICH may benefit from the exploration of cell therapies that target microglial phenotype switching, potentially improving patient outcomes. (There seems to be controversy regarding the M1/M2 classification of microglia, with some scholars arguing that such a classification is overly simplistic (45). However, this classification is widely used and still plays a role in understanding the functions of microglia in brain hemorrhage).

The NLRP3 inflammasome is crucial in microglia-mediated neuroinflammation after ICH and is predominantly expressed in microglia (34). Studies have shown that pre-treatment with epigallocatechin-3-gallate (EGCG) can reduce the levels of NLRP3 inflammasome in microglia following ICH and promote the transition of microglia from M1 phenotype to M2 phenotype (46). Xia et al. experimentally demonstrated that activation of sirtuin 1 (SIRT1), a sirtuin family member, suppresses NLRP3 inflammasome signaling in microglia during subarachnoid hemorrhage (SAH). Additionally, it facilitates microglia from M1 phenotype to M2 phenotype, which could be beneficial in mitigating neuroinflammation (47). Based on the hypothesis that both SAH and ICH are classified as hemorrhagic strokes, we speculate that the activation of SIRT1 may exhibit similar therapeutic effects in the context of ICH. Although neither of the two referenced studies directly addresses the connection between the NLRP3 inflammasome and microglial polarization towards M1 or M2 phenotypes, It is reasonable to propose that the NLRP3 inflammasome may play a role in regulating this phenotypic transition in microglia. However, due to the limited amount of literature on this topic, this area remains underexplored and represents one of our future research directions.

### 3.2 Astrocytes mediated neuroinflammation

Astrocytes, the most prevalent glial cells in the CNS, are essential for supporting neurons and maintaining brain homeostasis. In response to pathological conditions, astrocytes become activated, transforming into reactive astrocytes. This process involves cell hypertrophy and an increase in the release of neurotoxic factors, playing a critical role in the development of inflammation and neuronal damage within the central CNS (48, 49). Activated microglia possess the capacity to stimulate astrocyte activation, as well as modulate astrocyte proliferation and differentiation (34). Astrocytes can be categorized into A1 and A2 phenotypes. Much like microglia, astrocytes are capable of secreting mediators, either promoting inflammation or performing immunoregulatory functions, depending on their specific polarized phenotype (50, 51). A1 astrocytes play a pro-inflammatory role by secreting neurotoxins that induce neuronal death and the apoptosis of mature oligodendrocytes, while inhibiting processes essential for neuronal survival and synaptogenesis. In contrast, A2 astrocytes exhibit anti-inflammatory activities, upregulating anti-inflammatory genes to support neuronal survival and growth. They also contribute to glial scar formation, aid in clearing cellular debris, and promote the repair of the BBB, thus facilitating tissue recovery and regeneration following CNS injury (50, 52). (Similarly, the A1/A2 classification of astrocytes is also debated internationally, and a new consensus on the classification of astrocytes has yet to be reached (53). We hold a similar view on the A1/A2 classification of astrocytes as we do for the M1/M2 classification of microglia.) ICH triggers astrocyte activation, regulating the release of pro-inflammatory factors. Research indicates a significant increase in reactive astrocytes surrounding the hematoma within 12 to 48 hours post-ICH, suggesting their involvement in the early inflammatory response and potential contribution to both neuroprotection and neurotoxicity in the acute phase of injury (54, 55). After ICH, oxidative stress triggered by the release of hemoglobin activates astrocytes, leading to an increased expression of matrix metalloproteinase-9 (MMP-9). This contributes to the disruption of the BBB, increasing vascular permeability, which in turn exacerbates neuroinflammation and secondary brain injury (34). Following ICH, inhibiting the CD147 receptor on astrocytes has been shown to reduce MMP-9 levels in the surrounding hematoma, leading to decreased tissue degradation and improved neurological recovery. Additionally, enhancing the expression of aquaporin-4 (AQP4) on astrocytes may mitigate cerebral edema by regulating water homeostasis in the brain, thereby protecting against secondary injury and further functional decline. Therefore, modulating the activity of astrocytes may serve as a promising therapeutic approach to reduce neuroinflammation and cerebral edema following ICH. Modulating astrocyte function may help alleviate the progression of secondary brain injury and improve patient outcomes. Astrocytes also express the NLRP3 inflammasome (34). Within hypoxic-ischemic encephalopathy (HIE), transient receptor potential vanilloid 1 (TRPV1) intensifies astrocyte activation and promotes the secretion of IL-1β by astrocytes via the JAK2-STAT3 signaling pathway and activation of NLRP3 inflammasomes (56). Additionally, studies have shown that in rats with thalamic hemorrhage-induced central post-stroke pain (CPSP), around the thalamic injury area, the expression of NLRP3 is significantly increased with microglia and astrocytes activation. Thalamic administration of MCC950, a small-molecule NLRP3 inflammasome inhibitor, significantly diminishes microglia and astrocytes activation and lowers the secretion of pro-inflammatory cytokines (57). Neuroinflammation is characterized by an increase in the secretion of inflammatory mediators and microglia and astrocytes activation. The study’s findings highlight the critical interplay between astrocyte activation, the NLRP3 inflammasome, and neuroinflammation. However, research on the interaction between astrocyte activation, NLRP3 inflammasome activity, and neuroinflammation following ICH remains limited, which will be one of our future research focuses. Pyroptosis is a form of caspase-1-dependent programmed cell death, leading to the release of IL-1β and IL-18, which trigger a strong neuroinflammatory response. In a mouse model of ischemic stroke induced by middle cerebral artery occlusion (MCAO), the expression of lipocalin-2 (LCN2) in astrocytes markedly increases after 24 hours, which significantly promotes neuroinflammation. MCC950 inhibition of NLRP3 inflammasome activation reduces astrocyte pyroptosis, which is exacerbated by rLCN2 injection in mice (58). Both ICH and cerebral infarction are classified as strokes and exhibit similar pathological mechanisms. Therefore, exploring the link between astrocyte activation, NLRP3 inflammasome, and neuroinflammation in ICH could provide valuable insights for future therapeutic development.

## 4 The regulatory mechanisms of the NLRP3 inflammasome

**Figure 2:**
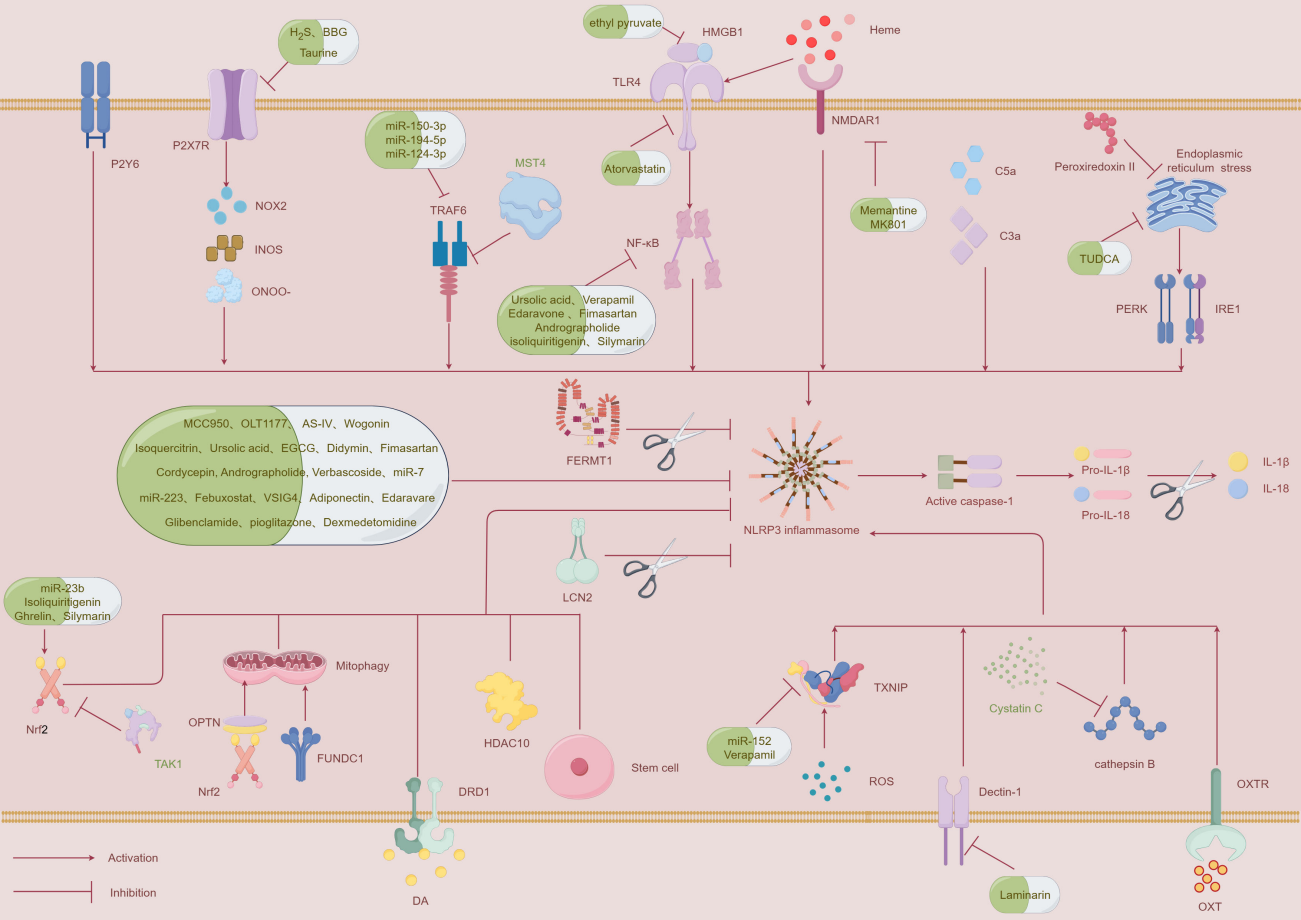
A schematic diagram illustrating the mechanisms that regulate the NLRP3 inflammasome and potential therapeutic strategies to inhibit its activation following ICH. This figure was created by Figdraw.

The NLRP3 inflammasome is regulated through multiple pathways, and this section highlights the latest insights into its regulation. In the following section, we will elaborate on the regulatory mechanisms involving ROS and thioredoxin-interacting protein (TXNIP), P2X7 receptor (P2X7R), tumor necrosis factor receptor-associated factor 6 (TRAF6), and nuclear factor erythroid-2 related factor 2 (Nrf2) (**Figure 2**, **Table 1**).

**Table 1:** Potential drugs for blocking NLRP3 inflammasome after ICH.

Targeted pathway	Drugs	Mechanism of action	Species	References
ROS/TXNIP/NLRP3 Pathway	miR-152	miR-152 effectively suppresses TXNIP expression, and its elevated levels prevent the binding of TXNIP to NLRP3, leading to a reduction in ROS generation	Rat	(89)
ROS/TXNIP/NLRP3 Pathway	verapamil	Reduces the secretion of TXNIP and NLRP3 around the hematoma area, inhibiting NF-kB activation	Mouse	(88)
P2X7R/NLRP3 Pathway	H_2_s	Directly scavenges ROS after ICH to inactivate NLRP3 inflammasome and suppress P2X7R expression	Rat	(93)
P2X7R/NLRP3 Pathway	BBG	Selective inhibitors of P2X7R have been shown to decrease the expression of NOX2, iNOS, and ONOO−, subsequently reducing the release of IL-1β, IL-18, and MPO	Rat	(94)
P2X7R/NLRP3 Pathway	Taurine	Upregulates H_2_S levels and reduces P2X7R expression	Rat	(95)
TRAF6/NLRP3 pathway	miR-150-3p	Overexpression of miR-150-3p inhibits the TRAF6/NLRP3 interaction, thereby suppressing NLRP3 inflammasome activation	Mouse	(100)
TRAF6/NLRP3 pathway	miR-194-5p	Overexpression of miR-194-5p reduces the TRAF6/NLRP3 interaction, thus inhibiting NLRP3 inflammasome activation	Rat	(98)
TRAF6/NLRP3 pathway	miR-124-3p	Targets TRAF6 expression and inhibits NLRP3 inflammasome activation	BV2	(97)
Nrf2/NLRP3 pathway	miR-23b	miR-23b decreases the binding of PTEN to NLRP3 and inhibits NLRP3 inflammasome activation	Rat	(106)
Nrf2/NLRP3 pathway	Isoliquiritigenin	Regulation of Nrf2 activity and its induced antioxidant system modulates ROS and/or NF-κB-mediated activation of the NLRP3 inflammasome pathway	Rat	(102)
Nrf2/NLRP3 pathway	Silymarin	Increases Nrf-2/HO-1 levels, inhibiting NF-κB and inflammasome-mediated expression of caspase-1 and IL-1β	Mouse	(101)
Nrf2/NLRP3 pathway	Ghrelin	Upregulates Nrf2 and downstream antioxidant gene expression, as well as NLRP3 inflammasome activation	Mouse	(104)
NMDAR1/NLRP3 pathway	Memantine	Inhibits nitric oxide synthase ser1412 phosphorylation through a mechanism related to NMDAR antagonism, subsequently reducing NLRP3 expression	Rat	(63)
NMDAR1/NLRP3 pathway	MK801	Reduces the levels of NLRP3 and IL-1β through antagonizing NMDAR	Mouse	(62)
ER stress/NLRP3 pathway	TUDCA	Inhibits the levels of NLRP3 and IL-13 by suppressing ER stress	Mouse	(73)
HMGB/TLR4/NLRP3	Ethyl pyruvate	Reduces the expression of NLRP3 inflammasome and IL-1β by inhibiting HMGB1	Rat	(76)
TLR4/NF-κB/NLRP3 pathway	Atorvastatin	Through the suppression of TLR4 and MyD88 upregulation, this mechanism reduces the levels of NLRP3 inflammasome	Mouse	(79)
Directly targeting the NLRP3 inflammasome	MCC950	Inhibits NLRP3 inflammasome activation and reduces IL-1β secretion	Mouse	(109)
Directly targeting the NLRP3 inflammasome	OLT1177	Inhibits the NLRP3 inflammasome activation and reduces the secretion of caspase-1 and IL-1β	Mouse	(15)
Directly targeting the NLRP3 inflammasome	AS-IV	Elevates KLF2 expression, allowing it to bind to the promoter region of NLRP3, thereby suppressing its transcription and subsequently reducing the levels of caspase-1, IL-1β, and IL-18	Mouse	(17)
Directly targeting the NLRP3 inflammasome	Wogonin	Inhibits METTL14-mediated NLRP3 methylation, thereby reducing the expression of NLRP3, caspase-1, and ASC	Mouse	(111)
Directly targeting the NLRP3 inflammasome	Isoquercitrin	Reduces the expression of the ion channel Piezo1, thereby regulating calcium ion influx and inhibiting NLRP3 expression	Rat	(113)
Directly targeting the NLRP3 inflammasome	Ursolic acid	Suppressing M1 polarization in microglia leads to a significant reduction in the levels of NLRP3, NF-κB, caspase-1, TNF-α, IL-6, and IL-1β	Rat	(9)
Directly targeting the NLRP3 inflammasome	EGCG	Promotes M2 polarization of microglia, upregulates HO-1 expression, and inhibits the levels of NLRP3, caspase-1, IL-1β, and IL-18	Mouse	(46)
Directly targeting the NLRP3 inflammasome	Didymin	Increases RKIP and binds to ASC to block NLRP3 inflammasome assembly, downregulating the levels of NLRP3, caspase-1, IL-1β, TNF-α	Mouse	(10)
Directly targeting the NLRP3 inflammasome	Cordycepin	Downregulates components of the NLRP3 inflammasome and reduces the release of IL-1β and IL-18	Mouse	(115)
Directly targeting the NLRP3 inflammasome	Andrographolide	Inhibits NLRP3 inflammasome assembly, suppresses NF-κB activation, and reduces IL-1β release	Rat	(116)
Directly targeting the NLRP3 inflammasome	Verbascoside	Inhibits NLRP3 expression and reduces microglial activation	Mouse	(117)
Directly targeting the NLRP3 inflammasome	miR-7	By inhibiting NLRP3 expression, this process decreases the production of TNF-α, IL-1β, IL-6, and caspase-1	Rat	(14)
Directly targeting the NLRP3 inflammasome	miR-223	Inhibits NLRP3 expression and reduces the expression of IL-1β and caspase-1	Mouse	(121)
Directly targeting the NLRP3 inflammasome	Febuxosta	Inhibits the expression of NLRP3 inflammasome, reducing the release of TNF-α and IL-1β	Mouse	(122)
Directly targeting the NLRP3 inflammasome	VSIG4	Increases the levels of phosphorylated JAK2 and STAT3 proteins as well as A20, and inhibits NLRP3 expression	Mouse	(123)
Directly targeting the NLRP3 inflammasome	Adiponectin	Inhibits NLRP3 expression and reduces the expression of IL-1β and IL-18	Rat	(124)
Directly targeting the NLRP3 inflammasome	Edaravone	Inhibits NF-κB-dependent NLRP3 in microglia, reducing the production of caspase-1 and IL-1β	Rat	(125)
Directly targeting the NLRP3 inflammasome	Glibenclamide	Inhibits NLRP3 inflammasome activation and reduces the production of IL-1β, IL-18, IL-6, and TNF-α	Mouse	(126)
Directly targeting the NLRP3 inflammasome	Pioglitazone	Increases lactate production through the inhibition of NLRP3 expression and enhancement of anaerobic glycolysis, thereby providing neuroprotection after brain hemorrhage	Mouse	(127)
Directly targeting the NLRP3 inflammasome	Dexmedetomidine	Increases lactate production through the inhibition of NLRP3 expression and enhancement of anaerobic glycolysis, thereby providing neuroprotection after brain hemorrhage	Mouse	(128)
Directly targeting the NLRP3 inflammasome	Fimasartan	Inhibits NLRP3 inflammasome and NF-κB activation	Rat	(130)
Dectin-1/NLRP3 pathway	Laminarin	Inhibits Dectin-1, reducing the levels of NLRP3, IL-1β, and IL-18	Mouse	(146)
BMSC/NLRP3 pathway	miR-183-5p	Reduces ROS levels and targets PDCD4 to inhibit NLRP3 inflammasome activation	Rat	(139)

### 4.1 Heme regulates the NLRP3 inflammasome

Upon erythrocyte destruction, substantial levels of hemoglobin are released into the extracellular space, where it undergoes oxidation. The oxidized hemoglobin then releases heme, a classical DAMP. When released from hemoproteins, heme acts as a potentially harmful molecule by catalyzing the production of reactive ROS (59, 60). Given that DAMPs and ROS are upstream signals for NLRP3 inflammasome activation, it suggests that heme may be involved in its activation. Hemin, derived from hemoglobin, becomes unstable at physiological temperatures after release. Ferrous heme (Fe2+) spontaneously oxidizes to ferric heme (Fe3+), dissociating from globin, and is classified as a DAMP. Earlier studies showed that in a mouse model of ICH with hemin injection, NLRP3 and IL-1β expression levels were significantly elevated. Moreover, the synergistic interaction with N-methyl-D-aspartate receptor 1 (NMDAR1) led to the activation of NLRP3, ultimately contributing to inflammatory damage (61, 62). Memantine, an antagonist of NMDAR, inhibits nitric oxide synthase ser^1412^ phosphorylation through an NMDAR-related antagonistic mechanism, thereby reducing peroxynitrite, MMP-9, and NLRP3 production, which in turn mitigates BBB disruption and neurological dysfunction (63). However, the precise mechanisms underlying NMDAR antagonism remain unclear, and this will be a focus of future research. Similarly, Weng et al. observed that administering the NMDAR antagonist MK801 reduces the expression of NLRP3 in heme-treated microglia (62). In a recent study, the injection of heme into the mouse striatum was shown to increase phosphorylation levels of extracellular signal-regulated kinase (ERK) and p38, indicating MAPK pathway activation associated with inflammation. Such activation triggers the secretion of pro-IL-1β, leading to lipid peroxidation and initiating an inflammatory response within the brain. Notably, the knockout of the NLRP3 gene in mice significantly mitigated behavioral impairments and reduced the release of inflammatory cytokines (64). These results imply that heme triggers neuroinflammation through a pathway reliant on NLRP3 activation. Lin et al. additionally found that in a mouse model of ICH, heme amplified microglial activation through TLR4, which in turn triggered NF-κB activation via the TLR4/MyD88/TRIF pathway, thus exacerbating inflammatory damage (65). The study did not thoroughly investigate whether the TLR4/MyD88/TRIF signaling pathway, following the activation of NF-κB, participates in the upregulation process of NLRP3. Given the upregulation of NLRP3 by NF-κB in the priming signals of inflammasome activation, we hypothesize that heme may promote the expression of NLRP3 through this pathway. Nevertheless, further research is required to validate this hypothesis. However, a previous study appears to contradict the conclusions of the aforementioned research. Tan et al. observed that while hemin-treated primary microglial cells could promote the activation of NLRP3 inflammasome and caspase-1, there was no corresponding increase in IL-1β secretion (61). Although the role of heme in activating the NLRP3 inflammasome has been relatively well-studied, the precise connection between NLRP3 activation and the subsequent secretion of inflammatory cytokines remains an unresolved issue in current research. Therefore, further investigation in future experiments is necessary to explore this relationship in greater detail. While collagenase and autologous blood injection are the standard methods for modeling ICH, the study suggests that heme injection may offer a novel model for exploring post-ICH neuroinflammation.

### 4.2 Dopamine regulates the NLRP3 inflammasome.

Dopamine is a monoamine neurotransmitter classified within the catecholamine family. It is essential for the regulation of motor function, behavior, endocrine responses, and cardiovascular activity, and acts as a critical link between the nervous and immune systems (66, 67). Yan et al. showed that dopamine regulates systemic inflammation through suppressing NLRP3 inflammasome activation in the hippocampus. Dopamine D1 receptor (DRD1) signaling mediates a negative regulation of hippocampal NLRP3 via the second messenger cyclic adenosine monophosphate (cAMP). This pathway promotes the ubiquitination of NLRP3 by the E3 ubiquitin ligase MARCH7, resulting in NLRP3 degradation and, consequently, controlling the inflammatory response both locally and systemically (67). In a mouse model of ICH, activation of DRD1 was found to suppress microglial activation and downregulate the expression of NLRP3, caspase-1, and IL-1β, while upregulating IFN-β. This modulation significantly reduced NLRP3-driven inflammatory responses, attenuated cerebral edema, and improved neurological outcomes (68). IFN-β is a well-known immunoregulatory cytokine, recognized for its potent anti-inflammatory effect. IFN-β inhibits NLRP3 inflammasome activation and promotes T cell polarization through STAT1 phosphorylation to exert these effects (69, 70).

However, the specific mechanism by which dopamine induces the upregulation of IFN-β was not addressed in the study. Investigating this regulatory pathway in future studies may provide valuable insights for more effectively managing inflammation associated with ICH.

### 4.3 Endoplasmic reticulum stress regulates the NLRP3 inflammasome

The endoplasmic reticulum (ER), a vital organelle within the cell, fulfills several essential functions, including lipid biosynthesis, promoting protein folding and maturation, and regulating intracellular calcium homeostasis. After ICH, ER stress is induced, which is closely associated with NLRP3 inflammasome activation (71). In obese mice, ER stress triggers the activation of key proteins such as protein kinase RNA-like ER kinase (PERK) and inositol-requiring enzyme-1α (IRE1α), which subsequently activate the NLRP3 inflammasome. This activation results in an increase in caspase-1 expression, which in turn triggers pyroptotic cell death (72). In summary, NLRP3 inflammasome activation induced by ER stress requires both PERK and IRE1 (71). Studies have shown that tauroursodeoxycholic acid (TUDCA) can inhibit ER stress, reducing NLRP3 inflammasome activation and the expression of inflammatory markers in the ICH model (73). Recent studies have also indicated that Peroxiredoxin II reduces neuroinflammation after intracerebral hemorrhage by alleviating neuronal pyroptosis and suppressing NLRP3 inflammasome activation through counteracting ER stress via the PI3K/AKT pathway (74). While research on ER stress-induced inflammasome activation in ICH remains limited, current evidence suggests that targeting ER stress could offer a promising therapeutic approach to reduce post-hemorrhagic inflammation.

### 4.4 TLR4 regulates the NLRP3 inflammasome

After ICH, high mobility group box 1 (HMGB1) is released from the nucleus into the cytoplasm, where it interacts with toll-like receptors (TLRs) on immune cells, initiating an inflammatory cascade. Experimental studies have demonstrated that knocking down HMGB1 or TLR4 significantly reduces associated neurological impairments (75). In a rat model of ICH, elevated expression of NLRP3 inflammasome and IL-1β were observed. Notably, inhibition of HMGB1 or TLR4 led to a substantial decrease in NLRP3 expression (76). Although this study did not address whether the HMGB1/TLR4 axis activates NF-κB expression, previous research has shown that intraperitoneal injection of ethyl pyruvate in diabetic ICH mice can reduce the level of NLRP3 inflammasome via the HMGB1/TLR4 pathway, while concurrently inhibiting NF-κB activation, thereby alleviating neuroinflammation (77). A series of studies have demonstrated that NLRP3, IL-1β are target genes of NF-κB (30, 78). Based on this evidence, we hypothesize that the HMGB1/TLR4 pathway promotes NLRP3 expression through NF-κB activation, exacerbating neuroinflammation following intracerebral hemorrhage. Although there is currently limited research on this pathway in the context of ICH, we hypothesize that targeting the HMGB1/TLR4 axis could serve as a potential therapeutic approach to mitigate post-ICH inflammation. Further studies are needed to validate this mechanism and its potential for improving post-ICH neurological outcomes. Similarly, another study indicated that the TLR4/MyD88 pathway can activate NF-κB, subsequently promoting NLRP3 expression. However, atorvastatin has been shown to interrupt this process, alleviating neuroinflammation following intracerebral hemorrhage (79). Taken together with the aforementioned findings, we hypothesize that directly or indirectly modulating TLR4 may serve as an effective approach for reducing neuroinflammation post-hemorrhage.

### 4.5 The other pathways regulating NLRP3 inflammasome

The P2Y6 receptor (P2Y6R), part of the P2Y purinergic receptor family, is extensively expressed in the nervous system. It is very important during a range of pathological processes, particularly in immune responses and inflammatory reactions (80, 81). Recent researches have indicated that following ICH, there is a significant increase in the levels of P2Y6R and NLRP3 inflammasome. P2Y6R exacerbates NLRP3-mediated cell apoptosis by activating the PI3K/AKT pathway. Furthermore, inhibition of P2Y6R decreases the expression of key inflammatory factors, highlighting its potential role in the regulation of neuroinflammation (82). Zhu et al. also discovered that a highly selective P2Y6R antagonist (compound 50) effectively suppressed NLRP3 inflammasome activation in intestinal tissues, thereby alleviating symptoms of ulcerative colitis (83). Given that both P2Y6R and P2X7R, as previously discussed, belong to the same purinergic receptor family, they may share similar mechanisms in regulating NLRP3 inflammasome activation. Alternatively, these two receptors could synergistically activate NLRP3 inflammasomes. Exploring this potential interaction may represent a promising future research direction. The development of a dual-target inhibitor capable of blocking both receptors could offer a novel therapeutic approach to mitigate neuroinflammation after ICH.

In rats with ICH, complement activation was first demonstrated, revealing its involvement in promoting brain edema and injury through multiple pathways (84). Complement is closely linked to inflammation in ICH, with the complement cascade representing the initial phase of sterile inflammation (85). Recent research has shown that during ICH-induced neuroinflammation, the levels of complement components C3a and C5a are elevated, and their supplementation can induce IL-1β expression. In contrast, inhibiting NLRP3 mitigates this effect, highlighting the crucial role of NLRP3 in complement-mediated neuroinflammation after ICH (86). However, studies in this area remain limited, and the precise mechanisms connecting complement activation with NLRP3 inflammasome activation have yet to be fully elucidated.

## 5 Potential interventions for NLRP3 inflammasome after ICH

The activation of the NLRP3 inflammasome is closely associated with the occurrence of neuroinflammation. Therefore, targeting the NLRP3 inflammasome is of great significance for alleviating neuroinflammation following intracerebral hemorrhage. Accordingly, we have outlined several effective therapeutic strategies targeting NLRP3 inflammasome activation(**Figure 2**, **Table 1**).

### 5.1 Targeted regulation of specific signaling pathways of the NLRP3 inflammasome

#### 5.1.1 Targeting ROS/TXNIP/NLRP3 pathway

Thioredoxin-interacting protein (TXNIP), as a critical component of the α-arrestin superfamily, plays an essential regulatory role in inflammation triggered by oxidative stress. TXNIP interacts with thioredoxin (TRX), and upon dissociation, it triggers NLRP3 inflammasome activation. Oxidative stress is a primary factor that disrupts the interaction between TXNIP and TRX, thereby activating the inflammasome (87, 88). Recent research has demonstrated that in thrombin-induced brain injury, thrombin enhances NLRP3 inflammasome expression via ROS/TXNIP signaling (87). Hu et al. additionally demonstrated that MicroRNA-152 can markedly inhibit TXNIP expression induced by ICH and disrupt the interaction between TXNIP and NLRP3. In microglial cells, overexpression of MicroRNA-152 reduced hemin-induced neuroinflammation and lowered ROS production, offering protective effects to co-cultured neurons. *In vivo*, overexpression of MicroRNA-152 in rat brains significantly mitigated neurological damage, cerebral edema, and promoted hematoma clearance following ICH (89). Verapamil, a commonly used phenylalkylamine L-type calcium (Ca2+) channel blocker (CCB), is pharmacologically recognized for its antihypertensive, anti-inflammatory, and BBB-modulating effects. Recent studies have demonstrated its benefits across various neurovascular conditions. In a collagenase-induced ICH mouse model, verapamil administration has demonstrated efficacy in reducing TXNIP and NLRP3 expression around the hematoma region, inhibiting NF-κB activation, facilitating hematoma clearance, and improving both neuroinflammation and blood-brain barrier dysfunction (88). Furthermore, in mouse models of hyperglycemic stroke, TXNIP promotes the activation of the NLRP3 inflammasome, thereby aggravating neuronal injury (90). Recent research indicates that in cerebral ischemia/reperfusion (I/R) injury, COG1410, an agonist of Low-density lipoprotein receptor-related protein-1 (LRP1), inhibits the TXNIP/NLRP3 signaling pathway and promotes the shift in microglial polarization from the M1 phenotype to the M2 phenotype, thereby contributing to anti-inflammatory effects (91). While the latter two studies do not focus specifically on ICH, an integrated analysis of these findings suggests that the ROS/TXNIP/NLRP3 pathway plays a significant pro-inflammatory role in cerebrovascular diseases. This suggests that further investigation into the inhibition of the ROS/TXNIP/NLRP3 pathway could be crucial for reducing neuroinflammation and improving outcomes following ICH.

#### 5.1.2 Targeting P2X7R/NLRP3 pathway

The P2X7 receptor (P2X7R) is a member of the P2X purinergic receptor family, which includes subtypes P2X1 to P2X7. P2X7R is a non-selective cation channel activated by ATP and is highly expressed in microglial cells and astrocytes (92, 93). Downregulation of the P2X7R gene effectively inhibits the NLRP3 inflammasome activation and reduces the secretion of IL-1 and IL-18, which helps to remarkably decrease brain edema and improve neurological impairments. Treatment with the specific P2X7R inhibitor Blue Brilliant G (BBG) has been shown to significantly reduce the levels of NADPH oxidase 2 (NOX2), inducible nitric oxide synthase (iNOS), and peroxynitrite (ONOO-), all of which are associated with neurotoxicity in the CNS following ICH. The application of peroxynitrite (ONOO-) decomposing catalysts significantly inhibits the NLRP3 inflammasome activation and reduces the release of IL-1β and IL-18 (94). Emerging research indicates that H_2_S offers considerable neuroprotection across a range of CNS conditions, including Alzheimer’s disease, subarachnoid hemorrhage, and ischemic stroke. After ICH, endogenous H_2_S levels decrease; however, exogenous supplementation of H_2_S can alleviate the P2X7R/NLRP3 inflammatory cascade, reducing brain edema, neurological deficits, and microglial accumulation following ICH (93). Moreover, a study suggested that high doses of taurine can mitigate post-ICH dysfunction, brain edema, and hematoma volume by upregulating H_2_S in brain tissue and downregulating P2X7R expression (95). Recent findings also showed that puerarin suppresses ferroptosis by inhibiting the P2X7R/NLRP3 pathway and mitigates Staphylococcus aureus-induced endometritis (96). While research on the P2X7R/NLRP3 pathway in the context of ICH is still limited, significant progress has been made in reducing inflammation via this pathway in other disease models. Therefore, developing more inhibitors or drugs targeting this signaling pathway will be one of our future research directions.

#### 5.1.3 Targeting TRAF6/NLRP3 pathway

Tumor necrosis factor receptor-associated factor 6 (TRAF6), a key adaptor protein in the TNF and TLR superfamilies, is well recognized for its strong connection to neural damage and NLRP3-driven inflammation. TRAF6 levels increase 24 hours post-ICH and bind to NLRP3, facilitating inflammasome activation (97, 98). Mammalian sterile 20-like kinase 4 (MST4), part of the GCK subfamily, functions as a key inhibitor of inflammation. MST4 alleviates the inflammatory response by directly phosphorylating TRAF6. Increased expression of the MST4 gene suppresses NLRP3 inflammasome activation following ICH, leading to a decrease in pro-inflammatory cytokine release, which in turn helps to reduce cerebral edema and improve neurological function (99). However, the study did not clarify the underlying mechanisms by which MST4 upregulation impacts TRAF6, leaving this question open for future investigation. Furthermore, the role of TRAF6 in regulating microglia-mediated secondary inflammatory responses post-ICH via NLRP3 inflammasome activation was not addressed. Consequently, exploring the interactions between TRAF6, NLRP3 inflammasome activation, and microglia-driven secondary neuroinflammation will be a primary focus in our future research endeavors.

In recent years, with the advancement of regenerative medicine, using stem cells and exosomes to promote neuroprotection and recovery after ICH has shown great potential. Sun et al. reported that exosomal miR-150-3p derived from stem cells can bind directly to TRAF6, exhibiting an inverse correlation with its expression levels. Mechanistically, exosomal miR-150-3p alleviates injury and neurological deficits after ICH by modulating the TRAF6/NF-κB axis (100). MST4, an anti-inflammatory regulator, directly phosphorylates the adaptor protein TRAF6, thereby inhibiting NLRP3 inflammasome activation. This process reduces neuroinflammation and helps alleviate brain injury after ICH. In contrast, Hesperadin, a selective inhibitor of MST4, increases NLRP3 expression, exacerbating neurological deficits and brain edema (99).Additionally, two microRNAs also regulate TRAF6 expression. miR-124-3p effectively suppresses the microglia-mediated secondary inflammatory response following ICH by modulating TRAF6 expression and inhibiting NLRP3 inflammasome activation (97). Likewise, the overexpression of miR-194-5p diminishes the interaction between TRAF6 and NLRP3, thereby suppressing NLRP3 inflammasome activation and alleviating neuroinflammation following ICH (98).

#### 5.1.4 Targeting Nrf2/NLRP3 pathway

Nuclear factor erythroid-2 related factor 2 (Nrf2) is a nuclear transcription factor that responds to oxidative stress by activating the expression of antioxidant and detoxifying enzymes, including NAD(P)H: quinone oxidoreductase-1 (NQO1) and catalase (CAT). This activation is essential for preserving intracellular homeostasis, as it regulates the redox balance within cells (101, 102). In animal models of ICH, Nrf2 is upregulated, which activates protective genes regulated by antioxidant response elements (AREs). This increase in expression suppresses reactive ROS production and inflammation, resulting in a neuroprotective effect (103). Previous researches have confirmed that Nrf2 primarily inhibits the NLRP3 inflammasome through reducing ROS levels and/or suppressing NF-κB activity, which helps alleviate early brain injury. In Nrf2 knockout animals, oxidative damage and leukocyte infiltration following ICH are significantly higher than in control groups (103, 104).

Transforming Growth Factor-beta-activated kinase 1 (TAK1), as a member of the MAP3K family within the mitogen-activated protein kinase (MAPK) cascade, plays a critical role in various disease models, especially in the generation of ROS and the amplification of oxidative damage. In a rat model of ICH, inhibiting TAK1 alleviated BBB disruption, neuroinflammation, and oxidative damage following ICH. Mechanistically, inhibition of TAK1 facilitates the nuclear translocation of NRF2, which in turn reduces ROS production and oxidative stress. This mechanism effectively suppresses NLRP3-mediated neuroinflammation, contributing to the mitigation of neuroinflammatory damage (105). Phosphatase and tensin homolog (PTEN) plays a crucial role in the regulation of oxidative stress and inflammation. PTEN can directly interact with NLRP3 and dephosphorylate it, thereby promoting the interaction between NLRP3 and ASC, which leads to inflammasome activation. In a rat with ICH, the intravenous injection of exosomal miR-23b derived from bone marrow mesenchymal stem cells (BMSCs) significantly reduced brain edema and led to improved behavioral outcomes. BMSC-exosomal miR-23b alleviates NLRP3-mediated neuroinflammation by promoting NRF2 nuclear translocation and inhibiting the interaction between PTEN and NLRP3 (106). Another study revealed that Nrf2 interacts with the mitophagy receptor optineurin (OPTN), regulating mitophagy to remove dysfunctional mitochondria following ICH. This interaction prevents the NLRP3 inflammasome activation, resulting in a reduction in neuroinflammation and mitigating secondary brain injury in rat models of ICH (103). Ghrelin has the ability to cross the BBB and provides neuroprotective effects in various CNS diseases, including subarachnoid hemorrhage and traumatic cerebral damage. In a mouse with ICH, intraperitoneal injection of Ghrelin reduced neuroinflammation, brain edema, and promoted hematoma clearance. Mechanistically, ghrelin activates the Nrf2/ARE pathway, enhancing antioxidant enzymes expression like NQO1. This results in reduced ROS levels and suppression of NLRP3 inflammasome activation (104). Zeng et al. also demonstrated that intraperitoneal injection of Isoliquiritigenin significantly inhibits the NF-κB and NLRP3 inflammasome pathways while activating the Nrf2-mediated antioxidant system, thereby alleviating cerebral edema, neuronal deformation, and neurological deficits following ICH (102). Silymarin has been shown to downregulate NF-κB expression, thereby inhibiting caspase-1 and IL-1β expression triggered by inflammasome activation. Additionally, it promotes the activation of the Nrf2/HO-1 signaling pathway. These lead to increased overall cellular protection and reduced inflammatory activation after ICH (101).

However, there is some controversy regarding whether Nrf2 can suppress NLRP3 expression. In several disease models, Nrf2 has been associated with NLRP3 inflammasome activation, promoting IL-1-mediated vascular inflammation and potentially aggravating conditions such as atherosclerosis (107). Recent researches reported that hydrogen gas can attenuate NLRP3 inflammasome activation through inhibiting Nrf2, which in turn reduces inflammation and helps alleviate sepsis-associated encephalopathy (108). It is possible that Nrf2 modulates NLRP3 via different mechanisms, either activating or inhibiting it, or that Nrf2 specifically inhibits NLRP3 expression in ICH models. Further studies are necessary to clarify this issue.

### 5.2 Directly targeting the NLRP3 inflammasome

#### 5.2.1 Inhibitor of the NLRP3 inflammasome

MCC950, a potent selective NLRP3 inflammasome inhibitor, effectively suppresses its activation even at nanomolar concentrations. In a mouse model of ICH, MCC950 significantly decreased IL-1β secretion, alleviated cerebral edema, and enhanced neurological recovery (109). A recent study further showed that in a mouse with ischemic stroke, MCC950 effectively lowered the levels of caspase-1 and IL-1β, leading to reduced brain edema and a smaller infarct area (110). A new NLRP3 inflammasome inhibitor, OLT1177, has recently emerged. In mice with intracerebral hemorrhage (ICH), treatment with OLT1177 significantly inhibited Caspase-1 activation and IL-1β release, notably reducing brain edema and improving neurological deficits following ICH (15). These results, along with earlier studies on MCC950, indicate that both OLT1177 and MCC950 may serve as promising therapeutic options for mitigating post-stroke neuroinflammation by inhibiting NLRP3 inflammasome activation.

#### 5.2.2 Inhibition of the NLRP3 inflammasome by traditional Chinese herbs and plant extracts

In recent years, traditional Chinese herbs and plant extracts have shown promising potential in mitigating neuroinflammation following ICH. Astragaloside IV(AS-IV), a monomer extracted from Radix Astragali, has demonstrated neuroprotective effects by modulating protein expression and reducing apoptosis, thereby alleviating neuroinflammation. In preclinical studies using a mouse model of ICH, AS-IV was found to inhibit NLRP3 inflammasome expression by upregulating Kruppel-like factor 2 (KLF2), which resulted in a reduction in brain edema, attenuation of neuroinflammation, and improvements in neurological function (17). Methylation of the NLRP3 gene is essential for regulating apoptosis in the context of ICH. NLRP3 mRNA methylation plays a crucial role in regulating cell apoptosis in brain hemorrhage. Wogonin, a monomer derived from traditional Chinese medicine, reduces pyroptosis following ICH by inhibiting the methyltransferase METTL14, which mediates NLRP3 m6A methylation. This action helps restore neurological function and decreases pro-inflammatory factor release (111). METTL14’s role in NLRP3 m6A methylation has demonstrated significant potential in regulating inflammasome activation. In an acute lung injury model, METTL14 promotes the activation of the NLRP3 inflammasome through NLRP3 mRNA m6A methylation, while METTL14 knockout alleviates injury (112). Targeting the methylation of NLRP3 may represent an important therapeutic approach to mitigate neuroinflammation following ICH. Isoquercitrin, found in functional foods such as mulberries, exerts its anti-inflammatory effects by scavenging reactive oxygen species, enhancing antioxidant enzyme activity, and inhibiting inflammatory factors. It can reduce the expression of the ion channel Piezo1, thereby modulating calcium influx and suppressing NLRP3 expression, ultimately alleviating neuronal damage following intracerebral hemorrhage in rats (113). Ursolic acid, a type of pentacyclic triterpene found in several plant species, is recognized for its potent anti-inflammatory properties. In a collagenase-induced rat ICH model, ursolic acid inhibits the transformation of microglia into the M1 phenotype. This was accompanied by a significant decrease in the levels of NF-κB, TNF-α, IL-6, IL-1β, and caspase-1. This reduction contributed to the alleviation of neuroinflammation following ICH. Mechanistically, ursolic acid exerts its neuroprotective effects primarily by inhibiting the NF-κB/NLRP3/GSDMD pathway (9). Bao et al. found that Epigallocatechin-3-gallate (EGCG) can reduce neuroinflammation after ICH by increasing the expression of heme oxygenase-1 (HO-1) and decreasing the levels of caspase-1, NLRP3, IL-1β, and IL-18. Additionally, EGCG enhances microglial polarization towards the M2 phenotype, which is known for its anti-inflammatory effects. This dual action of reducing inflammatory markers and promoting beneficial microglial phenotypes indicates that EGCG can effectively improve neuroinflammation after ICH (46). Didymin, a citrus-derived flavonoid, exhibits potent antioxidant, anti-inflammatory, and neuroprotective properties. Raf kinase inhibitor protein (RKIP) has been shown to bind to ASC, thereby preventing the assembly of the NLRP3 inflammasome. Didymin alleviates neuroinflammation following ICH by upregulating RKIP and downregulating the expression of NLRP3, caspase-1, IL-1β, TNF-α, and MPO (10, 114). Moreover, herbal compounds such as Cordycepin, Andrographolide, and Verbascoside have been shown to attenuate inflammation following ICH by inhibiting the NLRP3 inflammasome. Andrographolide can also inhibit the NF-κB signaling pathway (115–117). These studies suggest that traditional Chinese medicines and plant-derived extracts exhibit remarkable anti-inflammatory effects in the context of ICH. Future research into identifying additional herbal compounds or plant extracts capable of mitigating post-hemorrhagic inflammation holds significant therapeutic potential.

#### 5.2.3 Inhibition of the NLRP3 inflammasome by microRNA

MicroRNAs (miRNAs) are one of the most extensively studied classes of non-coding RNAs. They regulate gene expression by blocking mRNA translation and mediating mRNA degradation, playing significant roles in cell proliferation, apoptosis, differentiation, and metabolism (118–120). A recent study found that MicroRNA-7 is capable of inhibiting NLRP3 expression in microglia and macrophages. This action reduces both the protein and mRNA expression of pro-inflammatory cytokines, thereby offering protection to neurological function following ICH (14). Similarly, Yang et al. discovered that MicroRNA-223 binds to specific sites on NLRP3, leading to a reduction in its expression. This, in turn, decreases the expression of caspase-1 and IL-1β, ultimately alleviating cerebral edema and improving neurological function after ICH (121).

#### 5.2.4 Other direct pathways for inhibiting the NLRP3 inflammasome

Febuxostat, a xanthine oxidoreductase inhibitor, has been previously used in the treatment of chronic gout. In ICH mice pretreated with Febuxostat, inhibition of the NLRP3/ASC/caspase-1 pathway led to a reduction in neuronal deformation and cell death, thereby alleviating neurological deficits (122). V-set and immunoglobulin domain-containing 4 (VSIG4), which belongs to the B7 superfamily, is involved in transmitting inflammatory signals and has been implicated in various inflammatory conditions. Injection of VSIG4 into ICH mice can suppress NLRP3 expression through the JAK2-STAT3-A20 pathway, thereby reducing BBB disruption and cerebral edema (123). Adiponectin (APN) and Edaravone exert effects comparable to those of MCC950 through decreasing the levels of NLRP3, IL-1β, and IL-18, thus reducing brain injury and enhancing recovery following ICH. Edaravone can also inhibit the NF-κB signaling pathway (124, 125).Recently, two drugs commonly used for treating diabetes have also been applied to mitigate NLRP3-mediated neurological deficits following ICH. Glibenclamide has been shown to preserve the integrity of the BBB by inhibiting NLRP3 inflammasome activation in microvascular endothelial cells, thereby preventing ICH-associated damage (126). Meanwhile, pioglitazone exerts neuroprotective effects post-ICH by suppressing NLRP3 expression and enhancing anaerobic glycolysis, leading to increased lactate production and reduced cerebral edema (127).

Dexmedetomidine, a highly selective α2-adrenergic receptor agonist frequently employed for anesthesia, sedation, and analgesia, has also been found to downregulate NLRP3 expression following ICH. This promotes hematoma clearance, reduces cerebral edema, and alleviates neuroinflammation (128). Chronic hypertension is a leading risk factor for ICH, and fimasartan is commonly prescribed for managing this condition (129). Research has demonstrated that low doses of fimasartan significantly reduce NLRP3 inflammasome and NF-κB pathways activation, alleviating neuroinflammation and brain injury post-ICH without affecting blood pressure (130). Given that fimasartan’s antihypertensive effect is dose-dependent, future studies could investigate the potential impact of increasing fimasartan dosage on the prognosis of hypertensive ICH patients.

### 5.3 Other mechanisms inhibiting the NLRP3 inflammasome

Autophagy serves as a highly conserved cellular mechanism, playing an essential role in the removal of damaged, aging, or surplus cytoplasmic components, directing them to lysosomes for degradation and recycling. Mitophagy, a specialized subtype of autophagy, is tasked with the selective removal of impaired or malfunctioning mitochondria via lysosomal degradation. Emerging studies indicate that mitophagy helps to inhibit NLRP3 inflammasome activation, thereby regulating inflammatory responses (131, 132). FUN14 domain containing 1 (FUNDC1) is a key mitophagy receptor. Zheng and colleagues demonstrated that FUNDC1 mitigates neuroinflammation following ICH by promoting mitophagy, which in turn suppresses the expression of NLRP3 inflammasome (133). Studies have also found that Nrf2 interacts with the mitochondrial autophagy receptor optineurin (OPTN), regulating mitophagy to clear dysfunctional mitochondria following intracerebral hemorrhage (ICH). This process inhibits NLRP3 inflammasome activation, thereby reducing neuroinflammation (103).However, conflicting evidence exists, with earlier studies showing that TLR4 can activate microglia and exacerbate inflammatory damage in ICH through autophagy pathways (134). This suggests ongoing debate about the precise role of autophagy in regulating inflammation post-ICH. Despite this, numerous studies have indicated that both autophagy and mitophagy can attenuate inflammation after ICH (135). So further studies are needed to elucidate the detailed mechanisms underlying these effects.

Stem cell therapy offers significant promise for promoting neuroprotection and enhancing neurorecovery (136). BMSC possess multi-differentiation and self-renewal abilities, offering significant potential in treating neurological sequelae following ICH (137, 138). Extracellular vesicles derived from BMSC have demonstrated the potential to enhance neurological function, decrease brain edema, and mitigate neuroinflammation in diabetic ICH, primarily through the delivery of miR-183-5p. Mechanistically, miR-183-5p exerts its effects by targeting PDCD4 and suppressing the NLRP3 pathway (139). Furthermore, hypoxic preconditioning of mesenchymal stem cells has been shown to suppress microglial activation, decrease IL-1β and TNF-α levels, and downregulate NLRP3 and Caspase-1 expression, thereby reducing pyroptosis and neuroinflammation following ICH (140). At present, stem cell-based therapies for ICH are gaining significant attention, with various types of stem cells being explored, including mesenchymal stem cells, embryonic stem cells, hematopoietic stem cells, and neural stem cells (136). However, most studies on inhibiting neuroinflammation after ICH have concentrated on mesenchymal stem cells. In the future, we may explore the potential of different stem cell types in alleviating neuroinflammation following ICH.

We found that knocking out certain genes can also inhibit NLRP3 expression and thereby reduce neuroinflammation following ICH. Zhao et al. found that LCN2 knockout in an ICH rat model significantly diminished the secretion of inflammatory cytokines including IL-1β, IL-18, and TNF-α. Additionally, it reduced NLRP3 inflammasome expression, facilitated hematoma clearance, and alleviated brain edema and inflammatory cell infiltration (141).Additionally, the FERM domain-containing kindlin 1 (FERMT1) is an integrin-associated protein. Knocking out FERMT1 reduced brain edema after ICH, promoted hematoma clearance, and improved behavioral outcomes. FERMT1 knockout was also found to suppress NLRP3 inflammasome activation, leading to a reduction in the release of pro-inflammatory cytokines such as IL-1β and IL-18 (142). The commonly used gene editing technologies, such as CRISPR-Cas9, have developed rapidly, and successful clinical trials have already been conducted. For example, Lu et al. used CRISPR-Cas9 technology to knock out Programmed Cell Death Protein 1 (PD-1) on human T cells to treat patients with non-small cell lung cancer, demonstrating strong safety and feasibility (143). However, the clinical translation of gene editing technology still faces significant obstacles. The application of gene editing in humans raises ethical controversies, particularly in cases involving the editing of human germline cells, which could lead to severe ethical debates. Additionally, gene editing technologies carry the risk of off-target effects, where the editing tool may mistakenly cut non-target genes during DNA cleavage, potentially leading to the deletion of essential genes and causing severe consequences. Therefore, the effectiveness of specific gene knockout treatments for post-ICH neuroinflammation remains to be determined.

Studies have shown that cathepsin B (CTSB) is crucial in regulating NLRP3 expression. The activation of the CTSB/NLRP3 signaling pathway exacerbates neurological damage, especially in cases of hippocampal injury (144). In a study conducted on rats with ICH, Zhou et al. reported that Cystatin C effectively inhibits CTSB-mediated activation of the NLRP3 inflammasome within microglia, consequently mitigating secondary brain damage after ICH (16). Oxytocin (OXT), secreted by neurons in the hypothalamus, plays a multifunctional role by promoting parturition and exhibiting anti-inflammatory and antioxidant properties through the stimulation of oxytocin receptors (OXTR). Yang et al. found that following ICH, endogenous OXT levels were decreased, while the expression of OXTR was significantly upregulated. Intranasal administration of OXT has been shown to suppress the secretion of NLRP3, caspase-1, IL-1, and IL-18. Mechanistically, OXT exerts its neuroprotective effects and attenuates neuroinflammation after ICH through activation of the OXTR/p-PKA/DRP1 signaling pathway (145). Dendritic cell-associated C-type lectin-1 (Dectin-1) is a pattern recognition receptor known to initiate inflammatory responses. Research has shown that Dectin-1 expression significantly increases in microglia following ICH. Laminarin-mediated inhibition of Dectin-1 has been shown to downregulate the expression of NLRP3, IL-1, and IL-18. This results in a reduction of brain edema and a decrease in neurological damage after ICH, suggesting that targeting Dectin-1 could be a promising approach for alleviating neuroinflammation and improving outcomes following ICH (146). Protein tyrosine phosphatase non-receptor type 22 (PTPN22) has been linked to the development of several inflammatory disorders. It plays a role in enhancing NLRP3 inflammasome activation and promoting the release of IL-1β, contributing to the inflammatory response (147). Wang et al. demonstrated that histone deacetylase 10 (HDAC10) can mitigate neuroinflammation after ICH by blocking the interaction between PTPN22 and NLRP3. This inhibition prevents inflammasome activation and reduces the inflammatory damage that follows (148).

## 6 Clinical treatment of intracerebral hemorrhage and the translational potential of targeting the NLRP3 inflammasome to control neuroinflammation in ICH

Acute spontaneous ICH represents the most prevalent subtype among ICH types. Cases of acute ICH typically require prompt and aggressive management, commonly involving surgical intervention or pharmacological therapy (149, 150).

For patients with cerebellar hemorrhage and rapid deterioration due to brainstem compression, surgical hematoma evacuation is broadly recognized as the standard of care. In cases of supratentorial hemorrhage, early craniotomy (within 24 hours of onset) to remove the hematoma is considered a critical life-saving intervention (151, 152). Following ICH, blood degradation products can induce inflammatory meningitis and hydrocephalus. Ventriculostomy facilitates the rapid evacuation of hemorrhage from the ventricles, significantly enhancing survival rates in patients with deteriorating neurological status (153, 154). In China, minimally invasive hematoma decompression surgery, guided by non-contrast CT with catheter or probe placement, has become the standard approach. In developed countries, this technique has demonstrated better prognostic outcomes compared to craniotomy and conservative treatment in brain hemorrhage management (152, 155, 156).

Hypertension is a significant risk factor for ICH. Consequently, effective blood pressure control is essential during both the preoperative and postoperative periods. Consistent regulation of blood pressure can help prevent ICH. Furthermore, findings from an international clinical trial indicate that early reduction of systolic blood pressure to below 140 mmHg in patients with ICH can modestly improve functional outcomes (157, 158). Additionally, strong evidence suggests that maintaining blood pressure within the range of 130–140 mmHg within six hours of ICH onset can enhance clinical prognosis (159). Hemostatic therapy is widely applied in the management of ICH, with the primary goal of mitigating the early expansion of hematomas. For patients who present within 4 hours of ICH onset, recombinant factor VIIa (rFVIIa) serves as a key therapeutic option (160, 161). Additionally, a large-scale international trial investigating the treatment of ultra-acute primary ICH demonstrated that intravenous administration of tranexamic acid within 8 hours of ICH onset significantly reduced the rates of mortality and severe adverse events within 7 days compared to the placebo group, while also modestly limiting hematoma expansion (159). Furthermore, the reversal of anticoagulant and antiplatelet therapies constitutes a critical component of ICH management. Patients with a history of anticoagulant or antiplatelet use generally exhibit poorer prognoses, underscoring the importance of timely intervention. For those with prior antiplatelet therapy, platelet transfusion has been shown to improve clinical outcomes. In cases involving warfarin-related anticoagulation, reversal strategies include the administration of vitamin K, prothrombin complex concentrates, fresh frozen plasma, or rFVIIa (150, 162).

Drugs targeting neuroinflammation after brain hemorrhage are currently under development or undergoing clinical evaluation (55). Statins, for instance, have demonstrated the ability to inhibit microglial activation in the CNS, reduce ROS and MMP production, and suppress inflammatory progression (163). In a clinical trial on rosuvastatin, statin therapy was associated with a reduced mortality rate in brain hemorrhage patients (164). Furthermore, a Danish registry study indicated that statin therapy could lower the risk of brain hemorrhage (165). We mentioned earlier that, in preclinical animal models, atorvastatin was shown to suppress NLRP3 expression and neuroinflammation via the TLR4/MYD88 signaling pathway (79). Minocycline, a tetracycline-class drug, has demonstrated anti-inflammatory effects by inhibiting MMP production and promoting the secretion of neurotrophic factors (166). In a randomized, blinded clinical trial, high-dose administration of minocycline within 12 hours of ICH symptom onset significantly reduced MMP-9 levels compared to the placebo group, which helped to mitigate inflammation (167). Similarly, preclinical studies in SAH rat models have shown that minocycline suppresses NLRP3 inflammasome expression and reduces IL-1β release, thereby alleviating inflammation (168). Although direct evidence on minocycline’s role in suppressing NLRP3 inflammasome expression in ICH is limited, the shared pathophysiological mechanisms between ICH and SAH suggest its potential for clinical translation based on findings from both clinical and preclinical studies. As previously discussed, pioglitazone exerts neuroprotective effects and alleviates cerebral edema following ICH in mice by inhibiting NLRP3 expression and enhancing anaerobic glycolysis to increase lactate production (127). The anti-inflammatory drug pioglitazone is currently undergoing a Phase I clinical trial for ICH, although the related results appear to remain unpublished (55). In preclinical studies using animal models, glibenclamide has been shown to protect the integrity of the blood-brain barrier by inhibiting the activation of NLRP3 inflammasome in microvascular endothelial cells, thereby preventing ICH-induced damage (126). Clinical trials investigating the use of glibenclamide in ICH are currently recruiting participants (55). Hypertension is a major risk factor for ICH, and the use of antihypertensive drugs has proven therapeutic value both before and after surgery (157, 158). As discussed earlier, fimasartan, an antihypertensive agent, significantly suppresses the activation of the NLRP3 inflammasome and the NF-κB pathway in rat models of ICH at low doses, thereby reducing neuroinflammation and brain injury (130). Although clinical trials for fimasartan in ICH have not yet begun, its preclinical results suggest considerable potential for clinical application in the comprehensive treatment of ICH.

In this review, we have highlighted various drugs capable of directly or indirectly inhibiting NLRP3 inflammasome activation. Among them, several are currently in clinical trials and show promising prospects for clinical translation based on trial outcomes.

## 7 Conclusions and future directions

ICH remains a significant challenge due to its high incidence and mortality rates. Neuroinflammation following ICH exacerbates inflammatory injury, contributing to brain edema and increasing intracranial pressure, which may further lead to ischemic damage. Thus, targeting neuroinflammation post-ICH is crucial for improving patient outcomes. NLRP3 inflammasome activation is pivotal in this process, typically leading to microglial activation and the subsequent secretion of pro-inflammatory cytokines. NLRP3 is expressed in both microglia and astrocytes, cells that play a crucial role in mediating neuroinflammatory responses. Pro-inflammatory M1 microglia and A1 astrocytes drive inflammation, whereas anti-inflammatory M2 microglia and A2 astrocytes are involved in tissue repair. Numerous studies indicate that inhibiting NLRP3 in microglia significantly reduces inflammation, cerebral edema, and neurological deficits after ICH. Additionally, models of HIE and CPSP show a clear association between NLRP3 upregulation and astrocyte activation. While research focusing on astrocytic NLRP3 inhibition in ICH is limited, it is plausible that jointly targeting NLRP3 in both microglia and astrocytes could represent a novel approach to mitigating post-ICH neuroinflammation.

With ongoing research in this area, multiple pathways have been uncovered that directly or indirectly downregulate NLRP3 expression. Notably, the ROS/TXNIP/NLRP3, P2X7R/NLRP3, TRAF6/NLRP3, and Nrf2/NLRP3 pathways have been extensively characterized. Nevertheless, considerable opportunities remain to explore alternative mechanisms for inhibiting NLRP3. For example, the increasing focus on stem cell therapies suggests that we might investigate the potential of different stem cell types, in combination with exosome therapy, to mitigate post-ICH inflammation. Although the knockout of specific genes in animal models can indeed alleviate neuroinflammation following ICH, its clinical translational potential remains uncertain. Furthermore, it is critical to investigate how both established and novel pathways that inhibit NLRP3 could be leveraged to modulate the phenotypes of microglia and astrocytes, a topic that has received limited attention in current research.

In summary, several drugs previously discussed that inhibit NLRP3 inflammasome activation are now in clinical trials, with promising preliminary findings highlighting their potential for clinical application. Modulating NLRP3 to reduce neuroinflammation after ICH represents a promising therapeutic avenue and may pave the way for innovative strategies to manage post-hemorrhagic inflammation in the future.

## References

[1] Ren H Pan Y Wang D Hao H Han R Qi C. CD22 blockade modulates microglia activity to suppress neuroinflammation following intracerebral hemorrhage. Pharmacol Res. (2023) 196:106912. doi: 10.1016/j.phrs.2023.106912 37696483

[2] Yan XL Xu FY Ji JJ Song P Pei YQ He MJ. Activation of UCP2 by anethole trithione suppresses neuroinflammation after intracerebral hemorrhage. Acta Pharmacol Sin. (2022) 43:811–28. doi: 10.1038/s41401-021-00698-1 PMC8976076 34183754

[3] Dai S Wei J Zhang H Luo P Yang Y Jiang X. Intermittent fasting reduces neuroinflammation in intracerebral hemorrhage through the Sirt3/Nrf2/HO-1 pathway. J Neuroinflamm. (2022) 19:122. doi: 10.1186/s12974-022-02474-2 PMC9137193 35624490

[4] Bautista W Adelson PD Bicher N Themistocleous M Tsivgoulis G Chang JJ. Secondary mechanisms of injury and viable pathophysiological targets in intracerebral hemorrhage. Ther Adv Neurol Disord. (2021) 14:17562864211049208. doi: 10.1177/17562864211049208 34671423 PMC8521409

[5] Duan Z Zhou W He S Wang W Huang H Yi L. Intranasal delivery of curcumin nanoparticles improves neuroinflammation and neurological deficits in mice with intracerebral hemorrhage. Small Methods. (2024) 8:e2400304. doi: 10.1002/smtd.202400304 38577823

[6] Wang Y Tian M Tan J Pei X Lu C Xin Y. Irisin ameliorates neuroinflammation and neuronal apoptosis through integrin αVβ5/AMPK signaling pathway after intracerebral hemorrhage in mice. J Neuroinflamm. (2022) 19:82. doi: 10.1186/s12974-022-02438-6 PMC8988353 35392928

[7] Tschoe C Bushnell CD Duncan PW Alexander-Miller MA Wolfe SQ. Neuroinflammation after intracerebral hemorrhage and potential therapeutic targets. J Stroke. (2020) 22:29–46. doi: 10.5853/jos.2019.02236 32027790 PMC7005353

[8] Xia S Zheng Y Yan F Chen G. MicroRNAs modulate neuroinflammation after intracerebral hemorrhage: Prospects for new therapy. Front Immunol. (2022) 13:945860. doi: 10.3389/fimmu.2022.945860 36389834 PMC9665326

[9] Lei P Li Z Hua Q Song P Gao L Zhou L. Ursolic Acid Alleviates Neuroinflammation after Intracerebral Hemorrhage by Mediating Microglial Pyroptosis via the NF-κB/NLRP3/GSDMD Pathway. Int J Mol Sci. (2023) 24:14771. doi: 10.3390/ijms241914771 37834220 PMC10572659

[10] Gu L Sun M Li R Zhang X Tao Y Yuan Y. Didymin suppresses microglia pyroptosis and neuroinflammation through the Asc/Caspase-1/GSDMD pathway following experimental intracerebral hemorrhage. Front Immunol. (2022) 13:810582. doi: 10.3389/fimmu.2022.810582 35154128 PMC8828494

[11] Wu CH Shyue SK Hung TH Wen S Lin CC Chang CF. Genetic deletion or pharmacological inhibition of soluble epoxide hydrolase reduces brain damage and attenuates neuroinflammation after intracerebral hemorrhage. J Neuroinflamm. (2017) 14:230. doi: 10.1186/s12974-017-1005-4 PMC5702198 29178914

[12] Zhou Y Wang Y Wang J Anne Stetler R Yang QW. Inflammation in intracerebral hemorrhage: from mechanisms to clinical translation. Prog Neurobiol. (2014) 115:25–44. doi: 10.1016/j.pneurobio.2013.11.003 24291544

[13] Keep RF Hua Y Xi G. Intracerebral haemorrhage: mechanisms of injury and therapeutic targets. Lancet Neurol. (2012) 11:720–31. doi: 10.1016/S1474-4422(12)70104-7 PMC3884550 22698888

[14] Luo B Li L Song XD Chen HX Yun DB Wang L. MicroRNA-7 attenuates secondary brain injury following experimental intracerebral hemorrhage via inhibition of NLRP3. J Stroke Cerebrovasc Dis. (2024) 33:107670. doi: 10.1016/j.jstrokecerebrovasdis.2024.107670 38438086

[15] Fang M Xia F Wang J Wang C Teng B You S. The NLRP3 inhibitor, OLT1177 attenuates brain injury in experimental intracerebral hemorrhage. Int Immunopharmacol. (2024) 131:111869. doi: 10.1016/j.intimp.2024.111869 38492343

[16] Zhou Y Dong W Wang L Ren S Wei W Wu G. Cystatin C attenuates perihematomal secondary brain injury by inhibiting the cathepsin B/NLRP3 signaling pathway in a rat model of intracerebral hemorrhage. Mol Neurobiol. (2024) 61:9646–62. doi: 10.1007/s12035-024-04195-4 38676809

[17] Wu H Chen S You G Lei B Chen L Wu J. The mechanism of astragaloside IV in NOD-like receptor family pyrin domain containing 3 inflammasome-mediated pyroptosis after intracerebral hemorrhage. Curr Neurovasc Res. (2024) 21:74–85. doi: 10.2174/0115672026295640240212095049 38409729

[18] Guo H Callaway JB Ting JP. Inflammasomes: mechanism of action, role in disease, and therapeutics. Nat Med. (2015) 21:677–87. doi: 10.1038/nm.3893 PMC4519035 26121197

[19] Jo EK Kim JK Shin DM Sasakawa C. Molecular mechanisms regulating NLRP3 inflammasome activation. Cell Mol Immunol. (2016) 13:148–59. doi: 10.1038/cmi.2015.95 PMC4786634 26549800

[20] Swanson KV Deng M Ting JP. The NLRP3 inflammasome: molecular activation and regulation to therapeutics. Nat Rev Immunol. (2019) 19:477–89. doi: 10.1038/s41577-019-0165-0 PMC7807242 31036962

[21] Schroder K Tschopp J. The inflammasomes. Cell. (2010) 140:821–32. doi: 10.1016/j.cell.2010.01.040 20303873

[22] Liu D Zeng X Li X Mehta JL Wang X. Role of NLRP3 inflammasome in the pathogenesis of cardiovascular diseases. Basic Res Cardiol. (2018) 113:5. doi: 10.1007/s00395-017-0663-9 29224086

[23] Moossavi M Parsamanesh N Bahrami A Atkin SL Sahebkar A. Role of the NLRP3 inflammasome in cancer. Mol Cancer. (2018) 17:158. doi: 10.1186/s12943-018-0900-3 30447690 PMC6240225

[24] Shao BZ Xu ZQ Han BZ Su DF Liu C. NLRP3 inflammasome and its inhibitors: a review. Front Pharmacol. (2015) 6:262. doi: 10.3389/fphar.2015.00262 26594174 PMC4633676

[25] Luo Y Reis C Chen S. NLRP3 inflammasome in the pathophysiology of hemorrhagic stroke: A review. Curr Neuropharmacol. (2019) 17:582–9. doi: 10.2174/1570159X17666181227170053 PMC6712291 30592254

[26] Broz P Dixit VM. Inflammasomes: mechanism of assembly, regulation and signalling. Nat Rev Immunol. (2016) 16:407–20. doi: 10.1038/nri.2016.58 27291964

[27] Lamkanfi M Dixit VM. Mechanisms and functions of inflammasomes. Cell. (2014) 157:1013–22. doi: 10.1016/j.cell.2014.04.007 24855941

[28] Zhang L Tang Y Huang P Luo S She Z Peng H. Role of NLRP3 inflammasome in central nervous system diseases. Cell Biosci. (2024) 14:75. doi: 10.1186/s13578-024-01256-y 38849934 PMC11162045

[29] Paik S Kim JK Silwal P Sasakawa C Jo EK. An update on the regulatory mechanisms of NLRP3 inflammasome activation. Cell Mol Immunol. (2021) 18:1141–60. doi: 10.1038/s41423-021-00670-3 PMC8093260 33850310

[30] Bauernfeind FG Horvath G Stutz A Alnemri ES MacDonald K Speert D. Cutting edge: NF-kappaB activating pattern recognition and cytokine receptors license NLRP3 inflammasome activation by regulating NLRP3 expression. J Immunol. (2009) 183:787–91. doi: 10.4049/jimmunol.0901363 PMC2824855 19570822

[31] Ozaki E Campbell M Doyle SL. Targeting the NLRP3 inflammasome in chronic inflammatory diseases: current perspectives. J Inflammation Res. (2015) 8:15–27. doi: 10.2147/JIR.S51250 PMC4303395 25653548

[32] Rabeony H Pohin M Vasseur P Petit-Paris I Jegou JF Favot L. IMQ-induced skin inflammation in mice is dependent on IL-1R1 and MyD88 signaling but independent of the NLRP3 inflammasome. Eur J Immunol. (2015) 45:2847–57. doi: 10.1002/eji.2015.45.issue-10 26147228

[33] Kim EH Park MJ Park S Lee ES. Increased expression of the NLRP3 inflammasome components in patients with Behcet’s disease. J Inflammation (Lond). (2015) 12:41. doi: 10.1186/s12950-015-0086-z PMC4487834 26136643

[34] Xiao L Wang M Shi Y Xu Y Gao Y Zhang W. Secondary white matter injury mediated by neuroinflammation after intracerebral hemorrhage and promising therapeutic strategies of targeting the NLRP3 inflammasome. Curr Neuropharmacol. (2023) 21:669–86. doi: 10.2174/1570159X20666220830115018 PMC10207923 36043798

[35] Dutra FF Alves LS Rodrigues D Fernandez PL de Oliveira RB Golenbock DT. Hemolysis-induced lethality involves inflammasome activation by heme. Proc Natl Acad Sci U S A. (2014) 111:E4110–8. doi: 10.1073/pnas.1405023111 PMC4191786 25225402

[36] Ma Q Chen S Hu Q Feng H Zhang JH Tang J. NLRP3 inflammasome contributes to inflammation after intracerebral hemorrhage. Ann Neurol. (2014) 75:209–19. doi: 10.1002/ana.24070 PMC4386653 24273204

[37] Zhou X Zhao R Lv M Xu X Liu W Li X. ACSL4 promotes microglia-mediated neuroinflammation by regulating lipid metabolism and VGLL4 expression. Brain Behav Immun. (2023) 109:331–43. doi: 10.1016/j.bbi.2023.02.012 36791893

[38] Woodburn SC Bollinger JL Wohleb ES. The semantics of microglia activation: neuroinflammation, homeostasis, and stress. J Neuroinflamm. (2021) 18:258. doi: 10.1186/s12974-021-02309-6 PMC8571840 34742308

[39] Olude MA Mouihate A Mustapha OA Farina C Quintana FJ Olopade JO. Astrocytes and microglia in stress-induced neuroinflammation: the African perspective. Front Immunol. (2022) 13:795089. doi: 10.3389/fimmu.2022.795089 35707531 PMC9190229

[40] Yang R Yang B Liu W Tan C Chen H Wang X. Emerging role of non-coding RNAs in neuroinflammation mediated by microglia and astrocytes. J Neuroinflamm. (2023) 20:173. doi: 10.1186/s12974-023-02856-0 PMC10363317 37481642

[41] Kanazawa M Ninomiya I Hatakeyama M Takahashi T Shimohata T. Microglia and monocytes/macrophages polarization reveal novel therapeutic mechanism against stroke. Int J Mol Sci. (2017) 18:2135. doi: 10.3390/ijms18102135 29027964 PMC5666817

[42] Orihuela R McPherson CA Harry GJ. Microglial M1/M2 polarization and metabolic states. Br J Pharmacol. (2016) 173:649–65. doi: 10.1111/bph.v173.4 PMC4742299 25800044

[43] Hu X Leak RK Shi Y Suenaga J Gao Y Zheng P. Microglial and macrophage polarization-new prospects for brain repair. Nat Rev Neurol. (2015) 11:56–64. doi: 10.1038/nrneurol.2014.207 25385337 PMC4395497

[44] Kawabori M Kacimi R Kauppinen T Calosing C Kim JY Hsieh CL. Triggering receptor expressed on myeloid cells 2 (TREM2) deficiency attenuates phagocytic activities of microglia and exacerbates ischemic damage in experimental stroke. J Neurosci. (2015) 35:3384–96. doi: 10.1523/JNEUROSCI.2620-14.2015 PMC4339351 25716838

[45] Paolicelli RC Sierra A Stevens B Tremblay ME Aguzzi A Ajami B. Microglia states and nomenclature: A field at its crossroads. Neuron. (2022) 110:3458–83. doi: 10.1016/j.neuron.2022.10.020 PMC9999291 36327895

[46] Bao B Yin XP Wen XQ Suo YJ Chen ZY Li DL. The protective effects of EGCG was associated with HO-1 active and microglia pyroptosis inhibition in experimental intracerebral hemorrhage. Neurochem Int. (2023) 170:105603. doi: 10.1016/j.neuint.2023.105603 37633650

[47] Xia DY Yuan JL Jiang XC Qi M Lai NS Wu LY. SIRT1 promotes M2 microglia polarization via reducing ROS-mediated NLRP3 inflammasome signaling after subarachnoid hemorrhage. Front Immunol. (2021) 12:770744. doi: 10.3389/fimmu.2021.770744 34899720 PMC8653696

[48] Nakano-Kobayashi A Canela A Yoshihara T Hagiwara M. Astrocyte-targeting therapy rescues cognitive impairment caused by neuroinflammation via the Nrf2 pathway. Proc Natl Acad Sci U S A. (2023) 120:e2303809120. doi: 10.1073/pnas.2303809120 37549281 PMC10438385

[49] Singh D. Astrocytic and microglial cells as the modulators of neuroinflammation in Alzheimer’s disease. J Neuroinflamm. (2022) 19:206. doi: 10.1186/s12974-022-02565-0 PMC9382837 35978311

[50] Lawrence JM Schardien K Wigdahl B Nonnemacher MR. Roles of neuropathology-associated reactive astrocytes: a systematic review. Acta Neuropathol Commun. (2023) 11:42. doi: 10.1186/s40478-023-01526-9 36915214 PMC10009953

[51] Kwon HS Koh SH. Neuroinflammation in neurodegenerative disorders: the roles of microglia and astrocytes. Transl Neurodegener. (2020) 9:42. doi: 10.1186/s40035-020-00221-2 33239064 PMC7689983

[52] Fan YY Huo J. A1/A2 astrocytes in central nervous system injuries and diseases: Angels or devils? Neurochem Int. (2021) 148:105080. doi: 10.1016/j.neuint.2021.105080 34048845

[53] Escartin C Galea E Lakatos A O’Callaghan JP Petzold GC Serrano-Pozo A. Reactive astrocyte nomenclature, definitions, and future directions. Nat Neurosci. (2021) 24:312–25. doi: 10.1038/s41593-020-00783-4 PMC8007081 33589835

[54] Fei X Dou YN Wang L Wu X Huan Y Wu S. Homer1 promotes the conversion of A1 astrocytes to A2 astrocytes and improves the recovery of transgenic mice after intracerebral hemorrhage. J Neuroinflamm. (2022) 19:67. doi: 10.1186/s12974-022-02428-8 PMC8922810 35287697

[55] Xue M Yong VW. Neuroinflammation in intracerebral haemorrhage: immunotherapies with potential for translation. Lancet Neurol. (2020) 19:1023–32. doi: 10.1016/S1474-4422(20)30364-1 33212054

[56] Yang XL Wang X Shao L Jiang GT Min JW Mei XY. TRPV1 mediates astrocyte activation and interleukin-1beta release induced by hypoxic ischemia (HI). J Neuroinflamm. (2019) 16:114. doi: 10.1186/s12974-019-1487-3 PMC6540554 31142341

[57] Shi ZM Jing JJ Xue ZJ Chen WJ Tang YB Chen DJ. Stellate ganglion block ameliorated central post-stroke pain with comorbid anxiety and depression through inhibiting HIF-1alpha/NLRP3 signaling following thalamic hemorrhagic stroke. J Neuroinflamm. (2023) 20:82. doi: 10.1186/s12974-023-02765-2 PMC10031944 36944982

[58] Li J Xu P Hong Y Xie Y Peng M Sun R. Lipocalin-2-mediated astrocyte pyroptosis promotes neuroinflammatory injury via NLRP3 inflammasome activation in cerebral ischemia/reperfusion injury. J Neuroinflamm. (2023) 20:148. doi: 10.1186/s12974-023-02819-5 PMC10288712 37353794

[59] Salgar S Bolivar BE Flanagan JM Anum SJ Bouchier-Hayes L. The NLRP3 inflammasome fires up heme-induced inflammation in hemolytic conditions. Transl Res. (2023) 252:34–44. doi: 10.1016/j.trsl.2022.08.011 36041706 PMC10351365

[60] Bozza MT Jeney V. Pro-inflammatory actions of heme and other hemoglobin-derived DAMPs. Front Immunol. (2020) 11:1323. doi: 10.3389/fimmu.2020.01323 32695110 PMC7339442

[61] Tan Y Tan SW Fan BY Li L Zhou YG. Hemin induces the activation of NLRP3 inflammasome in N9 microglial cells. Iran J Immunol. (2018) 15:122–32. doi: 10.22034/iji.2018.39376 29947341

[62] Weng X Tan Y Chu X Wu XF Liu R Tian Y. N-methyl-D-aspartic acid receptor 1 (NMDAR1) aggravates secondary inflammatory damage induced by hemin-NLRP3 pathway after intracerebral hemorrhage. Chin J Traumatol. (2015) 18:254–8. doi: 10.1016/j.cjtee.2015.11.010 26777707

[63] Chen X Xiang X Xie T Chen Z Mou Y Gao Z. Memantine protects blood-brain barrier integrity and attenuates neurological deficits through inhibiting nitric oxide synthase ser1412 phosphorylation in intracerebral hemorrhage rats: involvement of peroxynitrite-related matrix metalloproteinase-9/NLRP3 inflammasome activation. Neuroreport. (2021) 32:228–37. doi: 10.1097/WNR.0000000000001577 PMC7870044 33470757

[64] Vasconcellos LRC Martimiano L Dantas DP Fonseca FM Mata-Santos H Travassos L. Intracerebral injection of heme induces lipid peroxidation, neuroinflammation, and sensorimotor deficits. Stroke. (2021) 52:1788–97. doi: 10.1161/STROKEAHA.120.031911 33827248

[65] Lin S Yin Q Zhong Q Lv FL Zhou Y Li JQ. Heme activates TLR4-mediated inflammatory injury via MyD88/TRIF signaling pathway in intracerebral hemorrhage. J Neuroinflamm. (2012) 9:46. doi: 10.1186/1742-2094-9-46 PMC3344687 22394415

[66] Possemato E La Barbera L Nobili A Krashia P D’Amelio M. The role of dopamine in NLRP3 inflammasome inhibition: Implications for neurodegenerative diseases. Ageing Res Rev. (2023) 87:101907. doi: 10.1016/j.arr.2023.101907 36893920

[67] Yan Y Jiang W Liu L Wang X Ding C Tian Z. Dopamine controls systemic inflammation through inhibition of NLRP3 inflammasome. Cell. (2015) 160:62–73. doi: 10.1016/j.cell.2014.11.047 25594175

[68] Wang T Nowrangi D Yu L Lu T Tang J Han B. Activation of dopamine D1 receptor decreased NLRP3-mediated inflammation in intracerebral hemorrhage mice. J Neuroinflamm. (2018) 15:2. doi: 10.1186/s12974-017-1039-7 PMC5753458 29301581

[69] Kasper LH Reder AT. Immunomodulatory activity of interferon-beta. Ann Clin Transl Neurol. (2014) 1:622–31. doi: 10.1002/acn3.84 PMC4184564 25356432

[70] Guarda G Braun M Staehli F Tardivel A Mattmann C Förster I. Type I interferon inhibits interleukin-1 production and inflammasome activation. Immunity. (2011) 34:213–23. doi: 10.1016/j.immuni.2011.02.006 21349431

[71] Mohammed Thangameeran SI Tsai ST Hung HY Hu WF Pang CY Chen SY. A role for endoplasmic reticulum stress in intracerebral hemorrhage. Cells. (2020) 9:750. doi: 10.3390/cells9030750 32204394 PMC7140640

[72] Lebeaupin C Proics E de Bieville CH Rousseau D Bonnafous S Patouraux S. ER stress induces NLRP3 inflammasome activation and hepatocyte death. Cell Death Dis. (2015) 6:e1879. doi: 10.1038/cddis.2015.248 26355342 PMC4650444

[73] Chen G Gao C Yan Y Wang T Luo C Zhang M. Inhibiting ER stress weakens neuronal pyroptosis in a mouse acute hemorrhagic stroke model. Mol Neurobiol. (2020) 57:5324–35. doi: 10.1007/s12035-020-02097-9 32880859

[74] Jin MH Liu XD Sun HN Han YH Kwon T. Peroxiredoxin II exerts neuroprotective effects by inhibiting endoplasmic reticulum stress and oxidative stress-induced neuronal pyroptosis. Mol Biol Rep. (2024) 51:607. doi: 10.1007/s11033-024-09568-5 38704801

[75] Lei C Li Y Zhu X Li H Chang X. HMGB1/TLR4 induces autophagy and promotes neuroinflammation after intracerebral hemorrhage. Brain Res. (2022) 1792:148003. doi: 10.1016/j.brainres.2022.148003 35820449

[76] Lei C Chen K Gu Y Li Y Wang L Zhu X. HMGB1/TLR4 axis promotes pyroptosis after ICH by activating the NLRP3 inflammasome. J Neuroimmunol. (2024) 393:578401. doi: 10.1016/j.jneuroim.2024.578401 38996718

[77] Wang Y Li K Liu Z Sun Y Wang J Liu Q. Ethyl pyruvate alleviating inflammatory response after diabetic cerebral hemorrhage. Curr Neurovasc Res. (2022) 19:196–202. doi: 10.2174/1567202619666220602153937 35657042

[78] Hiscott J Marois J Garoufalis J D’Addario M Roulston A Kwan I. Characterization of a functional NF-kappa B site in the human interleukin 1 beta promoter: evidence for a positive autoregulatory loop. Mol Cell Biol. (1993) 13:6231–40. doi: 10.1128/MCB.13.10.6231 PMC364682 8413223

[79] Chen D Sui L Chen C Liu S Sun X Guan J. Atorvastatin suppresses NLRP3 inflammasome activation in intracerebral hemorrhage via TLR4- and MyD88-dependent pathways. Aging (Albany NY). (2022) 14:462–76. doi: 10.18632/aging.203824 PMC8791214 35017318

[80] Anwar S Pons V Rivest S. Microglia purinoceptor P2Y6: an emerging therapeutic target in CNS diseases. Cells. (2020) 9:1595. doi: 10.3390/cells9071595 32630251 PMC7407337

[81] Liu GD Ding JQ Xiao Q Chen SD. P2Y6 receptor and immunoinflammation. Neurosci Bull. (2009) 25:161–4. doi: 10.1007/s12264-009-0120-3 PMC5552562 19448690

[82] Li Y Tu H Zhang S Ding Z Wu G Piao J. P2Y6 receptor activation aggravates NLRP3-dependent microglial pyroptosis via downregulation of the PI3K/AKT pathway in a mouse model of intracerebral hemorrhage. Mol Neurobiol. (2024) 61:4259–77. doi: 10.1007/s12035-023-03834-6 38079109

[83] Kira S Yoshiyama M Tsuchiya S Shigetomi E Miyamoto T Nakagomi H. P2Y(6)-deficiency increases micturition frequency and attenuates sustained contractility of the urinary bladder in mice. Sci Rep. (2017) 7:771. doi: 10.1038/s41598-017-00824-2 28396595 PMC5429706

[84] Holste K Xia F Garton HJL Wan S Hua Y Keep RF. The role of complement in brain injury following intracerebral hemorrhage: A review. Exp Neurol. (2021) 340:113654. doi: 10.1016/j.expneurol.2021.113654 33617886 PMC8119338

[85] Doyle SL Campbell M Ozaki E Salomon RG Mori A Kenna PF. NLRP3 has a protective role in age-related macular degeneration through the induction of IL-18 by drusen components. Nat Med. (2012) 18:791–8. doi: 10.1038/nm.2717 PMC3984677 22484808

[86] Yao ST Cao F Chen JL Chen W Fan RM Li G. NLRP3 is required for complement-mediated caspase-1 and IL-1beta activation in ICH. J Mol Neurosci. (2017) 61:385–95. doi: 10.1007/s12031-016-0874-9 27933491

[87] Ye X Zuo D Yu L Zhang L Tang J Cui C. ROS/TXNIP pathway contributes to thrombin induced NLRP3 inflammasome activation and cell apoptosis in microglia. Biochem Biophys Res Commun. (2017) 485:499–505. doi: 10.1016/j.bbrc.2017.02.019 28202418

[88] Ismael S Patrick D Salman M Parveen A Stanfill AG Ishrat T. Verapamil inhibits TXNIP-NLRP3 inflammasome activation and preserves functional recovery after intracerebral hemorrhage in mice. Neurochem Int. (2022) 161:105423. doi: 10.1016/j.neuint.2022.105423 36244583

[89] Hu L Zhang H Wang B Ao Q He Z. MicroRNA-152 attenuates neuroinflammation in intracerebral hemorrhage by inhibiting thioredoxin interacting protein (TXNIP)-mediated NLRP3 inflammasome activation. Int Immunopharmacol. (2020) 80:106141. doi: 10.1016/j.intimp.2019.106141 31982825

[90] Ismael S Nasoohi S Yoo A Ahmed HA Ishrat T. Tissue plasminogen activator promotes TXNIP-NLRP3 inflammasome activation after hyperglycemic stroke in mice. Mol Neurobiol. (2020) 57:2495–508. doi: 10.1007/s12035-020-01893-7 PMC9479162 32172516

[91] Yang CJ Li X Feng XQ Chen Y Feng JG Jia J. Activation of LRP1 ameliorates cerebral ischemia/reperfusion injury and cognitive decline by suppressing neuroinflammation and oxidative stress through TXNIP/NLRP3 signaling pathway in mice. Oxid Med Cell Longev. (2022) 2022:8729398. doi: 10.1155/2022/8729398 36035210 PMC9410841

[92] Zhao H Chen Y Feng H. P2X7 receptor-associated programmed cell death in the pathophysiology of hemorrhagic stroke. Curr Neuropharmacol. (2018) 16:1282–95. doi: 10.2174/1570159X16666180516094500 PMC6251042 29766811

[93] Zhao H Pan P Yang Y Ge H Chen W Qu J. Endogenous hydrogen sulphide attenuates NLRP3 inflammasome-mediated neuroinflammation by suppressing the P2X7 receptor after intracerebral haemorrhage in rats. J Neuroinflamm. (2017) 14:163. doi: 10.1186/s12974-017-0940-4 PMC5563049 28821266

[94] Feng L Chen Y Ding R Fu Z Yang S Deng X. P2X7R blockade prevents NLRP3 inflammasome activation and brain injury in a rat model of intracerebral hemorrhage: involvement of peroxynitrite. J Neuroinflamm. (2015) 12:190. doi: 10.1186/s12974-015-0409-2 PMC4609067 26475134

[95] Zhao H Qu J Li Q Cui M Wang J Zhang K. Taurine supplementation reduces neuroinflammation and protects against white matter injury after intracerebral hemorrhage in rats. Amino Acids. (2018) 50:439–51. doi: 10.1007/s00726-017-2529-8 29256178

[96] Yuan C Liu L Zhao Y Wang K. Puerarin inhibits Staphylococcus aureus-induced endometritis through attenuating inflammation and ferroptosis via regulating the P2X7/NLRP3 signalling pathway. J Cell Mol Med. (2024) 28:e18550. doi: 10.1111/jcmm.v28.14 39042561 PMC11265464

[97] Fang Y Hong X. miR-124-3p inhibits microglial secondary inflammation after basal ganglia hemorrhage by targeting TRAF6 and repressing the activation of NLRP3 inflammasome. Front Neurol. (2021) 12:653321. doi: 10.3389/fneur.2021.653321 34413820 PMC8369369

[98] Wan SY Li GS Tu C Chen WL Wang XW Wang YN. MicroNAR-194-5p hinders the activation of NLRP3 inflammasomes and alleviates neuroinflammation during intracerebral hemorrhage by blocking the interaction between TRAF6 and NLRP3. Brain Res. (2021) 1752:147228. doi: 10.1016/j.brainres.2020.147228 33385377

[99] Wu X Zhang Y Zhang Y Xia L Yang Y Wang P. MST4 attenuates NLRP3 inflammasome-mediated neuroinflammation and affects the prognosis after intracerebral hemorrhage in mice. Brain Res Bull. (2021) 177:31–8. doi: 10.1016/j.brainresbull.2021.09.006 34534636

[100] Sun J Xu G. Mesenchymal stem cell-derived exosomal miR-150-3p affects intracerebral hemorrhage by regulating TRAF6/NF-κB axis, gut microbiota and metabolism. Stem Cell Rev Rep. (2023) 19:1907–21. doi: 10.1007/s12015-023-10541-1 37099039

[101] Yuan R Fan H Cheng S Gao W Xu X Lv S. Silymarin prevents NLRP3 inflammasome activation and protects against intracerebral hemorrhage. BioMed Pharmacother. (2017) 93:308–15. doi: 10.1016/j.biopha.2017.06.018 28651232

[102] Zeng J Chen Y Ding R Feng L Fu Z Yang S. Isoliquiritigenin alleviates early brain injury after experimental intracerebral hemorrhage via suppressing ROS- and/or NF-κB-mediated NLRP3 inflammasome activation by promoting Nrf2 antioxidant pathway. J Neuroinflamm. (2017) 14:119. doi: 10.1186/s12974-017-0895-5 PMC5470182 28610608

[103] Cheng Y Liu M Tang H Chen B Yang G Zhao W. iTRAQ-based quantitative proteomics indicated Nrf2/OPTN-mediated mitophagy inhibits NLRP3 inflammasome activation after intracerebral hemorrhage. Oxid Med Cell Longev. (2021) 2021:6630281. doi: 10.1155/2021/6630281 33628368 PMC7892225

[104] Cheng Y Chen B Xie W Chen Z Yang G Cai Y. Ghrelin attenuates secondary brain injury following intracerebral hemorrhage by inhibiting NLRP3 inflammasome activation and promoting Nrf2/ARE signaling pathway in mice. Int Immunopharmacol. (2020) 79:106180. doi: 10.1016/j.intimp.2019.106180 31926478

[105] Zhao J Chen C Ge L Jiang Z Hu Z Yin L. TAK1 inhibition mitigates intracerebral hemorrhage-induced brain injury through reduction of oxidative stress and neuronal pyroptosis via the NRF2 signaling pathway. Front Immunol. (2024) 15:1386780. doi: 10.3389/fimmu.2024.1386780 38756773 PMC11096530

[106] Hu LT Wang BY Fan YH He ZY Zheng WX. Exosomal miR-23b from bone marrow mesenchymal stem cells alleviates oxidative stress and pyroptosis after intracerebral hemorrhage. Neural Regener Res. (2023) 18:560–7. doi: 10.4103/1673-5374.346551 PMC9727431 36018178

[107] Freigang S Ampenberger F Spohn G Heer S Shamshiev AT Kisielow J. Nrf2 is essential for cholesterol crystal-induced inflammasome activation and exacerbation of atherosclerosis. Eur J Immunol. (2011) 41:2040–51. doi: 10.1002/eji.201041316 21484785

[108] Xie K Zhang Y Wang Y Meng X Wang Y Yu Y. Hydrogen attenuates sepsis-associated encephalopathy by NRF2 mediated NLRP3 pathway inactivation. Inflammation Res. (2020) 69:697–710. doi: 10.1007/s00011-020-01347-9 32350570

[109] Ren H Kong Y Liu Z Zang D Yang X Wood K. Selective NLRP3 (Pyrin domain-containing protein 3) inflammasome inhibitor reduces brain injury after intracerebral hemorrhage. Stroke. (2018) 49:184–92. doi: 10.1161/STROKEAHA.117.018904 PMC5753818 29212744

[110] Ismael S Zhao L Nasoohi S Ishrat T. Inhibition of the NLRP3-inflammasome as a potential approach for neuroprotection after stroke. Sci Rep. (2018) 8:5971. doi: 10.1038/s41598-018-24350-x 29654318 PMC5899150

[111] Li L Gong J Zhang W. Treatment of intracerebral hemorrhage with traditional Chinese medicine monomer wogonin by modifying NLRP3 with METTL14 to inhibit neuronal cell pyroptosis. Appl Biochem Biotechnol. (2024) 196:6174–88. doi: 10.1007/s12010-023-04849-4 38224394

[112] Cao F Chen G Xu Y Wang X Tang X Zhang W. METTL14 contributes to acute lung injury by stabilizing NLRP3 expression in an IGF2BP2-dependent manner. Cell Death Dis. (2024) 15:43. doi: 10.1038/s41419-023-06407-6 38218935 PMC10787837

[113] Guo T Chen G Yang L Deng J Pan Y. Piezo1 inhibitor isoquercitrin rescues neural impairment mediated by NLRP3 after intracerebral hemorrhage. Exp Neurol. (2024) 379:114852. doi: 10.1016/j.expneurol.2024.114852 38857751

[114] Gu L Sun M Li R Tao Y Luo X Xu J. Activation of RKIP binding ASC attenuates neuronal pyroptosis and brain injury via caspase-1/GSDMD signaling pathway after intracerebral hemorrhage in mice. Transl Stroke Res. (2022) 13:1037–54. doi: 10.1007/s12975-022-01009-4 35355228

[115] Cheng Y Wei Y Yang W Song Y Shang H Cai Y. Cordycepin confers neuroprotection in mice models of intracerebral hemorrhage via suppressing NLRP3 inflammasome activation. Metab Brain Dis. (2017) 32:1133–45. doi: 10.1007/s11011-017-0003-7 28401330

[116] Li X Wang T Zhang D Li H Shen H Ding X. Andrographolide ameliorates intracerebral hemorrhage induced secondary brain injury by inhibiting neuroinflammation induction. Neuropharmacology. (2018) 141:305–15. doi: 10.1016/j.neuropharm.2018.09.015 30218674

[117] Zhou H Zhang C Huang C. Verbascoside attenuates acute inflammatory injury caused by an intracerebral hemorrhage through the suppression of NLRP3. Neurochem Res. (2021) 46:770–7. doi: 10.1007/s11064-020-03206-9 33400023

[118] Ul Hussain M. Micro-RNAs (miRNAs): genomic organisation, biogenesis and mode of action. Cell Tissue Res. (2012) 349:405–13. doi: 10.1007/s00441-012-1438-0 22622804

[119] Großhans H Chatterjee S. MicroRNases and the regulated degradation of mature animal miRNAs. Adv Exp Med Biol. (2011) 700:140–55. doi: 10.1007/978-1-4419-7823-3_12 21755479

[120] Cabarcas SM Thomas S Zhang X Cherry JM Sebastian T Yerramilli S. The role of upregulated miRNAs and the identification of novel mRNA targets in prostatospheres. Genomics. (2012) 99:108–17. doi: 10.1016/j.ygeno.2011.11.007 PMC3430075 22206861

[121] Yang Z Zhong L Xian R Yuan B. MicroRNA-223 regulates inflammation and brain injury via feedback to NLRP3 inflammasome after intracerebral hemorrhage. Mol Immunol. (2015) 65:267–76. doi: 10.1016/j.molimm.2014.12.018 25710917

[122] Bai Y Shi H Zhang Y Zhang C Wu B Wu X. Febuxostat attenuates secondary brain injury caused by cerebral hemorrhage through inhibiting inflammatory pathways. Iran J Basic Med Sci. (2024) 27:740–6. doi: 10.22038/IJBMS.2024.74655.16212 PMC11024405 38645501

[123] Ji N Wu L Shi H Li Q Yu A Yang Z. VSIG4 Attenuates NLRP3 and Ameliorates Neuroinflammation via JAK2-STAT3-A20 Pathway after Intracerebral Hemorrhage in Mice. Neurotox Res. (2022) 40:78–88. doi: 10.1007/s12640-021-00456-5 35013905

[124] Wang S Yao Q Wan Y Wang J Huang C Li D. Adiponectin reduces brain injury after intracerebral hemorrhage by reducing NLRP3 inflammasome expression. Int J Neurosci. (2020) 130:301–8. doi: 10.1080/00207454.2019.1679810 31607194

[125] Miao H Jiang Y Geng J Zhang B Zhu G Tang J. Edaravone Administration Confers Neuroprotection after Experimental Intracerebral Hemorrhage in Rats via NLRP3 Suppression. J Stroke Cerebrovasc Dis. (2020) 29:104468. doi: 10.1016/j.jstrokecerebrovasdis.2019.104468 31694784

[126] Xu F Shen G Su Z He Z Yuan L. Glibenclamide ameliorates the disrupted blood-brain barrier in experimental intracerebral hemorrhage by inhibiting the activation of NLRP3 inflammasome. Brain Behav. (2019) 9:e01254. doi: 10.1002/brb3.2019.9.issue-4 30859754 PMC6456786

[127] Kim H Lee JE Yoo HJ Sung JH Yang SH. Effect of pioglitazone on perihematomal edema in intracerebral hemorrhage mouse model by regulating NLRP3 expression and energy metabolism. J Korean Neurosurg Soc. (2020) 63:689–97. doi: 10.3340/jkns.2020.0056 PMC7671775 33105536

[128] Song HL Zhang SB. Therapeutic effect of dexmedetomidine on intracerebral hemorrhage via regulating NLRP3. Eur Rev Med Pharmacol Sci. (2019) 23:2612–9. doi: 10.26355/eurrev_201903_17411 30964190

[129] Saavedra JM. Angiotensin II AT(1) receptor blockers as treatments for inflammatory brain disorders. Clin Sci (Lond). (2012) 123:567–90. doi: 10.1042/CS20120078 PMC3501743 22827472

[130] Yang X Sun J Kim TJ Kim YJ Ko SB Kim CK. Pretreatment with low-dose fimasartan ameliorates NLRP3 inflammasome-mediated neuroinflammation and brain injury after intracerebral hemorrhage. Exp Neurol. (2018) 310:22–32. doi: 10.1016/j.expneurol.2018.08.013 30171865 PMC6203658

[131] Bravo-San-Pedro JM Kroemer G Galluzzi L. Autophagy and mitophagy in cardiovascular disease. Circ Res. (2017) 120:1812–24. doi: 10.1161/CIRCRESAHA.117.311082 28546358

[132] Moyzis AG Sadoshima J Gustafsson ÅB. Mending a broken heart: the role of mitophagy in cardioprotection. Am J Physiol Heart Circ Physiol. (2015) 308:H183–92. doi: 10.1152/ajpheart.00708.2014 PMC4312945 25437922

[133] Zheng S Jian D Gan H Wang L Zhao J Zhai X. FUNDC1 inhibits NLRP3-mediated inflammation after intracerebral hemorrhage by promoting mitophagy in mice. Neurosci Lett. (2021) 756:135967. doi: 10.1016/j.neulet.2021.135967 34022268

[134] Yang Z Liu B Zhong L Shen H Lin C Lin L. Toll-like receptor-4-mediated autophagy contributes to microglial activation and inflammatory injury in mouse models of intracerebral haemorrhage. Neuropathol Appl Neurobiol. (2015) 41:e95–106. doi: 10.1111/nan.2015.41.issue-4 25185720

[135] Fu K Xu W Lenahan C Mo Y Wen J Deng T. Autophagy regulates inflammation in intracerebral hemorrhage: Enemy or friend? Front Cell Neurosci. (2022) 16:1036313. doi: 10.3389/fncel.2022.1036313 36726453 PMC9884704

[136] Gao L Xu W Li T Chen J Shao A Yan F. Stem cell therapy: A promising therapeutic method for intracerebral hemorrhage. Cell Transpl. (2018) 27:1809–24. doi: 10.1177/0963689718773363 PMC6300771 29871521

[137] Ma Y Qi M An Y Zhang L Yang R Doro DH. Autophagy controls mesenchymal stem cell properties and senescence during bone aging. Aging Cell. (2018) 17:e12709. doi: 10.1111/acel.2018.17.issue-1 29210174 PMC5770781

[138] Vaquero J Otero L Bonilla C Aguayo C Rico MA Rodriguez A. Cell therapy with bone marrow stromal cells after intracerebral hemorrhage: impact of platelet-rich plasma scaffolds. Cytotherapy. (2013) 15:33–43. doi: 10.1016/j.jcyt.2012.10.005 23260084

[139] Ding H Jia Y Lv H Chang W Liu F Wang D. Extracellular vesicles derived from bone marrow mesenchymal stem cells alleviate neuroinflammation after diabetic intracerebral hemorrhage via the miR-183-5p/PDCD4/NLRP3 pathway. J Endocrinol Invest. (2021) 44:2685–98. doi: 10.1007/s40618-021-01583-8 34024028

[140] Liu J He J Huang Y Ge L Xiao H Zeng L. Hypoxia-preconditioned mesenchymal stem cells attenuate microglial pyroptosis after intracerebral hemorrhage. Ann Transl Med. (2021) 9:1362. doi: 10.21037/atm-21-2590 34733914 PMC8506532

[141] Zhao Y Xiao Q Sun T Yu H Luo M. Knockdown of LCN2 attenuates brain injury after intracerebral hemorrhage via suppressing pyroptosis. Neuropsychiatr Dis Treat. (2024) 20:83–99. doi: 10.2147/NDT.S440065 38249526 PMC10800110

[142] Wei J Yin J Cui Y Wang K Hong M Cui J. FERM domain containing kindlin 1 knockdown attenuates inflammation induced by intracerebral hemorrhage in rats via NLR family pyrin domain containing 3/nuclear factor kappa B pathway. Exp Anim. (2023) 72:324–35. doi: 10.1538/expanim.22-0145 PMC10435358 36740252

[143] Lu Y Xue J Deng T Zhou X Yu K Deng L. Safety and feasibility of CRISPR-edited T cells in patients with refractory non-small-cell lung cancer. Nat Med. (2020) 26:732–40. doi: 10.1038/s41591-020-0840-5 32341578

[144] Zhang H Wang J Ruan C Gao Z Zhu Q Li S. Co-exposure of chronic stress and alumina nanoparticles aggravates hippocampal microglia pyroptosis by activating cathepsin B/NLRP3 signaling pathway. J Hazard Mater. (2022) 436:129093. doi: 10.1016/j.jhazmat.2022.129093 35569374

[145] Yang M Deng S Jiang J Tian M Xiao L Gong Y. Oxytocin improves intracerebral hemorrhage outcomes by suppressing neuronal pyroptosis and mitochondrial fission. Stroke. (2023) 54:1888–900. doi: 10.1161/STROKEAHA.123.043391 37317879

[146] Ding Z Zhong Z Wang J Zhang R Shao J Li Y. Inhibition of dectin-1 alleviates neuroinflammatory injury by attenuating NLRP3 inflammasome-mediated pyroptosis after intracerebral hemorrhage in mice: preliminary study results. J Inflammation Res. (2022) 15:5917–33. doi: 10.2147/JIR.S384020 PMC9579968 36274828

[147] Spalinger MR Kasper S Gottier C Lang S Atrott K Vavricka SR. NLRP3 tyrosine phosphorylation is controlled by protein tyrosine phosphatase PTPN22. J Clin Invest. (2023) 133:4388. doi: 10.1172/JCI169304 PMC9927928 36787260

[148] Wang L Zheng S Zhang L Xiao H Gan H Chen H. Histone deacetylation 10 alleviates inflammation after intracerebral hemorrhage via the PTPN22/NLRP3 pathway in rats. Neuroscience. (2020) 432:247–59. doi: 10.1016/j.neuroscience.2020.02.027 32112918

[149] Schrag M Kirshner H. Management of intracerebral hemorrhage: JACC focus seminar. J Am Coll Cardiol. (2020) 75:1819–31. doi: 10.1016/j.jacc.2019.10.066 32299594

[150] Cordonnier C Demchuk A Ziai W Anderson CS. Intracerebral haemorrhage: current approaches to acute management. Lancet. (2018) 392:1257–68. doi: 10.1016/S0140-6736(18)31878-6 30319113

[151] Luparello V Canavero S. Treatment of hypertensive cerebellar hemorrhage–surgical or conservative management? Neurosurgery. (1995) 37:552–3. doi: 10.1227/00006123-199509000-00037 7501127

[152] Mendelow AD Gregson BA Fernandes HM Murray GD Teasdale GM Hope DT. Early surgery versus initial conservative treatment in patients with spontaneous supratentorial intracerebral haematomas in the International Surgical Trial in Intracerebral Haemorrhage (STICH): a randomised trial. Lancet. (2005) 365:387–97. doi: 10.1016/S0140-6736(05)70233-6 15680453

[153] Phan TG Koh M Vierkant RA Wijdicks EF. Hydrocephalus is a determinant of early mortality in putaminal hemorrhage. Stroke. (2000) 31:2157–62. doi: 10.1161/01.STR.31.9.2157 10978045

[154] Hemphill JC 3rd Greenberg SM Anderson CS Becker K Bendok BR Cushman M. Guidelines for the management of spontaneous intracerebral hemorrhage: A guideline for healthcare professionals from the American Heart Association/American Stroke Association. Stroke. (2015) 46:2032–60. doi: 10.1161/STR.0000000000000069 26022637

[155] Li Y Zhang H Wang X She L Yan Z Zhang N. Neuroendoscopic surgery versus external ventricular drainage alone or with intraventricular fibrinolysis for intraventricular hemorrhage secondary to spontaneous supratentorial hemorrhage: a systematic review and meta-analysis. PloS One. (2013) 8:e80599. doi: 10.1371/journal.pone.0080599 24232672 PMC3827437

[156] Zhou X Chen J Li Q Ren G Yao G Liu M. Minimally invasive surgery for spontaneous supratentorial intracerebral hemorrhage: a meta-analysis of randomized controlled trials. Stroke. (2012) 43:2923–30. doi: 10.1161/STROKEAHA.112.667535 22989500

[157] Anderson CS Heeley E Huang Y Wang J Stapf C Delcourt C. Rapid blood-pressure lowering in patients with acute intracerebral hemorrhage. N Engl J Med. (2013) 368:2355–65. doi: 10.1056/NEJMoa1214609 23713578

[158] Magid-Bernstein J Girard R Polster S Srinath A Romanos S Awad IA. Cerebral hemorrhage: pathophysiology, treatment, and future directions. Circ Res. (2022) 130:1204–29. doi: 10.1161/CIRCRESAHA.121.319949 PMC10032582 35420918

[159] Sprigg N Flaherty K Appleton JP Al-Shahi-Salman R Bereczki D Beridze M. Tranexamic acid for hyperacute primary IntraCerebral Haemorrhage (TICH-2): an international randomised, placebo-controlled, phase 3 superiority trial. Lancet. (2018) 391:2107–15. doi: 10.1016/S0140-6736(18)31033-X PMC5976950 29778325

[160] Mayer SA Brun NC Begtrup K Broderick J Davis S Diringer MN. Recombinant activated factor VII for acute intracerebral hemorrhage. N Engl J Med. (2005) 352:777–85. doi: 10.1056/NEJMoa042991 15728810

[161] Mayer SA Brun NC Begtrup K Broderick J Davis S Diringer MN. Efficacy and safety of recombinant activated factor VII for acute intracerebral hemorrhage. N Engl J Med. (2008) 358:2127–37. doi: 10.1056/NEJMoa0707534 18480205

[162] Qureshi AI Mendelow AD Hanley DF. Intracerebral haemorrhage. Lancet. (2009) 373:1632–44. doi: 10.1016/S0140-6736(09)60371-8 PMC3138486 19427958

[163] Arefieva TI Filatova AY Potekhina AV Shchinova AM. Immunotropic effects and proposed mechanism of action for 3-hydroxy-3-methylglutaryl-coenzyme A reductase inhibitors (Statins). Biochem (Mosc). (2018) 83:874–89. doi: 10.1134/S0006297918080023 30208827

[164] Tapia-Perez H Sanchez-Aguilar M Torres-Corzo JG Rodriguez-Leyva I Gonzalez-Aguirre D Gordillo-Moscoso A. Use of statins for the treatment of spontaneous intracerebral hemorrhage: results of a pilot study. Cent Eur Neurosurg. (2009) 70:15–20. doi: 10.1055/s-0028-1082064 19197830

[165] Ribe AR Vestergaard CH Vestergaard M Fenger-Grøn M Pedersen HS Lietzen LW. Statins and risk of intracerebral haemorrhage in a stroke-free population: A nationwide Danish propensity score matched cohort study. EClinicalMedicine. (2019) 8:78–84. doi: 10.1016/j.eclinm.2019.02.007 31193616 PMC6537517

[166] Yong VW Wells J Giuliani F Casha S Power C Metz LM. The promise of minocycline in neurology. Lancet Neurol. (2004) 3:744–51. doi: 10.1016/S1474-4422(04)00937-8 15556807

[167] Chang JJ Kim-Tenser M Emanuel BA Jones GM Chapple K Alikhani A. Minocycline and matrix metalloproteinase inhibition in acute intracerebral hemorrhage: a pilot study. Eur J Neurol. (2017) 24:1384–91. doi: 10.1111/ene.2017.24.issue-11 28929560

[168] Li J Chen J Mo H Chen J Qian C Yan F. Minocycline protects against NLRP3 inflammasome-induced inflammation and P53-associated apoptosis in early brain injury after subarachnoid hemorrhage. Mol Neurobiol. (2016) 53:2668–78. doi: 10.1007/s12035-015-9318-8 26143258

